# Linking root length and surface area to yield: variety-specific root plasticity in winter wheat across contrasting European environments

**DOI:** 10.1093/aob/mcaf155

**Published:** 2025-07-16

**Authors:** F Durand-Maniclas, H Heinemann, F Seidel, F Ciulla, T G de la Bárcena, M Camenzind, S Corrado, Z Csűrös, Zs Czakó, D Eylenbosch, A Ficke, C Flamm, J M Herrera, V Horáková, A Hund, F Lüddeke, F Platz, B Poós, D P Rasse, M da Silva-Lopes, M Toleikienė, A Veršulienė, M Visse-Mansiaux, K Yu, A Don, J Hirte

**Affiliations:** Agroscope, Agroecology and Environment, Soil Quality and Soil Use, 8046 Zurich, Switzerland; Thünen Institute, Institute of Climate-Smart Agriculture, 38116 Braunschweig, Germany; Thünen Institute, Institute of Climate-Smart Agriculture, 38116 Braunschweig, Germany; Agroscope, Agroecology and Environment, Soil Quality and Soil Use, 8046 Zurich, Switzerland; ETH Zurich, Institute of Agricultural Sciences, 8092 Zurich, Switzerland; Norwegian Institute of Bioeconomy Research (NIBIO), Biogeochemistry and Soil Quality, 1430 Ås, Norway; Technical University Munich (TUM), School of Life Sciences, Precision Agriculture Lab, 85354 Freising, Germany; ETH Zurich, Institute of Agricultural Sciences, 8092 Zurich, Switzerland; National Food Chain Safety Office (Nébih), Agricultural Genetic Resources Directorate, 1024 Budapest, Hungary; National Food Chain Safety Office (Nébih), Agricultural Genetic Resources Directorate, 1024 Budapest, Hungary; Walloon Agricultural Research Centre (CRAW), Crop Production Unit, 5030 Gembloux, Belgium; Norwegian Institute of Bioeconomy Research (NIBIO), Biogeochemistry and Soil Quality, 1430 Ås, Norway; Austrian Agency for Health and Food Safety (AGES), Seed/Seedling and Variety Assessment, 1220 Vienna, Austria; Agroscope, Plant Production Systems, Cultivation Techniques and Varieties in Arable Farming, 1260 Nyon, Switzerland; Central Institute for Supervising and Testing in Agriculture (ÚKZÚZ), Plant Production Section, National Plant Variety Office, 60300 Brno, Czech Republic; ETH Zurich, Institute of Agricultural Sciences, 8092 Zurich, Switzerland; Bundessortenamt, Test Management, Biochemical, Biophysical and Molecular Variety Testing, 30627 Hannover, Germany; Austrian Agency for Health and Food Safety (AGES), Seed/Seedling and Variety Assessment, 1220 Vienna, Austria; National Food Chain Safety Office (Nébih), Agricultural Genetic Resources Directorate, 1024 Budapest, Hungary; Norwegian Institute of Bioeconomy Research (NIBIO), Biogeochemistry and Soil Quality, 1430 Ås, Norway; Institute of Agrifood Research and Technology (IRTA), Plant Production, Sustainable Field Crops, 25194 Lleida, Spain; Lithuanian Research Center for Agriculture and Forestry (LAMMC), Institute of Agriculture, 58344 Kėdainiai, Lithuania; Lithuanian Research Center for Agriculture and Forestry (LAMMC), Institute of Agriculture, 58344 Kėdainiai, Lithuania; Agroscope, Plant Production Systems, Cultivation Techniques and Varieties in Arable Farming, 1260 Nyon, Switzerland; Technical University Munich (TUM), School of Life Sciences, Precision Agriculture Lab, 85354 Freising, Germany; Thünen Institute, Institute of Climate-Smart Agriculture, 38116 Braunschweig, Germany; Agroscope, Agroecology and Environment, Soil Quality and Soil Use, 8046 Zurich, Switzerland

**Keywords:** EJP Soil, *Triticum aestivum* L, winter wheat, root length, root surface area, heritability, pedoclimatic gradient, root plasticity, deep-rooting, yield, soil, management

## Abstract

**Background and aims:**

Understanding the relationship of root traits and crop performance under varying environmental conditions facilitates the exploitation of root characteristics in breeding and variety testing to maintain crop yields under climate change. Therefore, we (1) evaluated differences in root length and surface area between ten winter wheat varieties grown at 11 sites in Europe covering a large pedoclimatic gradient, (2) quantified differences in root response to soil, climate and management conditions between varieties, and (3) evaluated variety-specific relationships of grain yield and root length and surface area under diverse environmental conditions.

**Methods:**

At each site, we sampled the roots to 1 m soil depth after harvest and determined various root traits by scanning and image analysis. The impacts of soil, climate and management on roots and yield of the ten varieties were analysed by means of multivariate mixed models.

**Key results:**

Root length averaged 1.4 m root piece^−1^, 5007 m root m^−2^ soil, and 5300 m root m^−2^ soil and root surface area 0.039 m^2^ root piece^−1^, 40 m^2^ root m^−2^ soil, and 43 m^2^ root m^−2^ soil in 0.00–0.15 m, 0.15–0.50 m, 0.50–1.00 m soil depth, respectively. The variation in both traits was 10 times higher between sites than varieties, the latter ranging by a factor of 2 within sites. Irrespective of variety, temperature was a major driver of subsoil root traits, suggesting that warmer climates promoted root growth in deeper soil layers. Other soil and climate variables affected root length and/or root surface area of individual varieties, highlighting different degrees of root plasticity. The varieties displayed distinctly different relationships between yield and root traits under varying pedoclimatic conditions, highlighting genetic differences in yield response to environmentally driven root plasticity.

**Conclusions:**

These findings suggest that breeding efforts should target flexible root–yield relationships in the subsoil to maintain crop performance under climate change.

## INTRODUCTION

Climate change impacts are stressing agricultural production and increasingly hinder efforts to meet the demands of human nutrition (Nelson *et al.*, 2024 ). Since 1990, the rate of global warming per decade has consistently exceeded the average rate observed during the period from 1973 to 2022 and precipitation patterns have been shifting, with extreme weather events becoming more common - wetter regions getting wetter and drier regions getting drier (Giorgi *et al.*, 2004; Madsen *et al.*, 2014; Samset *et al.*, 2023 ). Rising temperatures lead to increased evapotranspiration and thereby increased risk of water shortage (Ajjur and Al-Ghamdi, 2021), while water shortage due to reduced precipitation can lead to increasing temperatures as a result of less evaporative cooling (Solomon *et al.*, 2007). As a consequence, global maize and wheat production have declined by 3.8 and 5.5 %, respectively (Lobell *et al.*, 2011; Moore and Lobell, 2015), and the yields of almost all major crops have stagnated since the mid-1990s in Southern Europe (Moore and Lobell, 2015; Agnolucci and De Lipsis, 2020; Le Gouis *et al.*, 2020; Brás *et al.*, 2021; Lopes, 2022; Gulino *et al.*, 2023). Crops that are adapted to extreme conditions such as drought may better cope with the effects of climate change. An important aspect of climate change adaptation in crops is closely linked to the characteristics of their root system as it facilitates the exploration and uptake of soil resources and thereby sustains vital physiological processes under abiotic stress (Gowda *et al.*, 2011; Lynch, 2013, 2018).

Root system architecture encompasses the spatial arrangement and attributes of root tissue within the soil profile and is commonly described by length, length density, volume, surface area, diameter, number of tips, branching frequency, and orientation of roots (Khan *et al.*, 2016). Higher root length and greater root surface area in deep soil are beneficial for accessing water in greater soil depths and can therefore better sustain yields during physiologically critical times of water shortage (Lynch, 2013; Li *et al.*, 2019; Maqbool *et al.*, 2022). Deep rooting also promotes organic carbon inputs in the subsoil (Kell, 2011; Lynch and Wojciechowski, 2015), which not only facilitates long-term carbon storage in agroecosystems (Paustian *et al.*, 2016) but also improves growth conditions for deep roots of succeeding crops (Rasse and Smucker, 1998). Understanding the impact of the root system on crop performance could facilitate the exploitation and manipulation of root characteristics to both increase crop yield and optimize agricultural land use (Smith and De Smet, 2012). The Food and Agriculture Organization (FAO) has therefore called for the inclusion of root traits in breeding programmes (FAO, 2013). However, this requires an understanding of root traits and their relationship to yield under varying environmental conditions (Colombo *et al.*, 2022).

The main drivers of root characteristics are attributed to genetics, environment and management (Rogers and Benfey, 2015; Hecht *et al.*, 2016). Different plant species exhibit distinct types of root systems, most notably tap or fibrous systems, and genotypes of a species develop certain root traits more strongly than others (Lynch, 1995; Osmont *et al.*, 2007; Fry *et al.*, 2018; Akman, 2020; Duan *et al.*, 2023). For instance, total root length was found to vary by factors of 2–4 among wheat genotypes (Adeleke *et al.*, 2020; Pariyar *et al.*, 2021), and by factors of 3–5 among maize genotypes (Hund *et al.*, 2007; Sun *et al.*, 2025). Environmental conditions directly affect the root system, as temperature, moisture and soil physical, chemical and biological properties influence all root traits (Rich and Watt, 2013), while agricultural management has an indirect effect by altering soil conditions. For example, root length of wheat can vary by a factor of 2 between sites and root depth by a factor of 1.2 between fertilization treatments on the same site (Svoboda *et al.*, 2020). The extent to which root traits respond to environmental conditions is expressed as root plasticity (Karlova *et al.*, 2021), which differs between crop varieties (Grossman and Rice, 2012), resulting in distinct genotype-by-environment interactions.

Wheat is globally one of the most important staple food crops (Shewry and Hey, 2015) and subject to intensive breeding efforts towards higher and more stable yields by increasing the harvest index (Siddique *et al.*, 1989) or adapting varieties to regionally specific biotic and abiotic stresses (IWGSC *et al.*, 2018). Root system architecture traits are currently not the priority of breeding targets or variety testing programmes, presumably due to their inherent hidden nature (Ober *et al.*, 2021), and selecting for these traits using only the shoot phenotype remains challenging (Severini *et al.*, 2020; Uga, 2021). Further, linking root traits to specific genes could enable targeted variety selection (Li *et al.*, 2021) but results between studies are mostly inconsistent (Alahmad *et al.*, 2019; Xu *et al.*, 2021; Chen *et al.*, 2022). Even single traits can be controlled by many different chromosomal regions and genes and even interactions between them (Raffo and Jensen, 2023). Few studies have focused on root trait variability in variety testing panels and their findings were confined to only a few growth environments (Mathew *et al.*, 2019; Fradgley *et al.*, 2020).

Therefore, the main objective of the present study was to investigate the relationship between yield and root traits of different winter wheat varieties under varying environmental conditions. Specifically, we (1) evaluated differences in root length and surface area between ten winter wheat varieties grown at 11 pedoclimatically diverse sites in Europe, (2) quantified differences in root response to pedoclimatic and management conditions between varieties, and (3) evaluated variety-specific relationships between grain yield and root length and surface area under diverse environmental conditions.

## MATERIALS AND METHODS

### Sites and wheat varieties

The study was conducted during the 2021–22 winter wheat season as part of a multi-location field experiment established 1 year earlier. The winter wheat trials were located at 11 sites (Table 1; Supplementary Data Fig. S1), which covered all major European pedoclimatic regions from the Mediterranean to the Boreal and from the Atlantic to the Pannonian zone (EEA, 2017). Nine sites had been established within the Horizon 2020 project INVITE and were assessed for above-ground crop performance by the INVITE partners in the wheat seasons 2020–21 and 2021–22 (Visse-Mansiaux et al., unpubl. res.): Grossnondorf AT (AT-Gn), Gembloux BE (BE-Ge), Changins CH (CH-Ca), Eschikon CH (CH-Es), Chrlice CZ (CZ-Ch), Freising DE (DE-Fr), Nossen DE (DE-No), Lleida ES (ES-Le) and Szekkutas HU (HU-Sz). To expand the pedoclimatic gradient to Northern Europe, two sites were added for the Horizon 2020 EJP Soil project MaxRoot-C in the exact same set-up as the INVITE sites in the 2021–22 wheat season: Dotnuva LT (LT-Do) and Ås NO (NO-As). The field designs corresponded to either randomized complete block, lattice, split block or latinized alpha designs according to national conventionality in variety testing. The plot size varied from 8.8 m^2^ to 19.2 m^2^ among sites, except for CH-Es (4 m^2^). The sites were managed according to local agricultural practices (Table 2) and phenological dates varied based on climatic differences between regions (Supplementary Data Fig. S2).

**Table 1.** Site characteristics: location, soil type and climate for 11 sites in Europe.

Country (institution^1^)	Site/ nearest town	Site abbreviation	Coordinates	Soil type^2^	Climate (Köppen-Geiger^3^)	MAT^4^ (°C)	Temperature September 2021 to August 2022 (°C)	MAP^4^ (mm)	Precipitation September 2021 to August 2022 (mm)
Austria (AGES)	Grossnondorf/Hollabrunn	AT-Gn	48°37'47.9″N 15°58'48.1″E	Calcaric Phaeozem	Dfb	10.2	11.2	650	422
Belgium (CRAW)	Gembloux	BE-Ge	50°35'52.0″N 4°41'24.5″E	Hortic Luvisol	Cfb	10.2	11.2	793	554
Switzerland (AGS)	Changins/Nyon	CH-Ca	46°24′03.6″N 6°13′55.1″E	Calcaric Cambisol	Dfb	10.7	12.4	995	692
Switzerland (ETH)	Eschikon/Lindau	CH-Es	47°27′02.3″N 8°40′56.4″E	Gleyic Cambisol	Cfb	9.2	10.9	1175	797
Czech Republic (UKZUZ)	Chrlice/Brno	CZ-Cr	49°7′28.99″N 16°38′03.0″E	Fluvisol	Dfb	9.0	11.6	612	451
Germany (TUM)	Dürnast/Freising	DE-Fr	48°24′25.4″N 11°41′39.1″E	Cambisol	Dfb	9.8	9.8	960	650
Germany (BSA)	Nossen	DE-No	51°3′20.02″N 13°16′31.7″E	Planosol	Dfb	9.2	10.5	645	474
Spain (IRTA)	Sucs/Lleida	ES-Le	41°41′44.7″N 0°25′35.1″E	Gypsisol	Cfb	15.5	13.3 ^5^	450	156 ^5^
Hungary (NEBIH)	Székkutas/Hódmezövásárhely	HU-Sz	46°30′45.3″N 20°31′15.3″E	Clayic Chernozem	Dfa	12.2	11.2	635	483
Lithuania (LAMMC)	Akademija/Dotnuva	LT-Do	55°23′28.6″N 23°51′49.8″E	Haplic Endocalcaric Luvisol	Dfb	7.8	8.0	705	686
Norway (NIBIO)	Ås	NO-As	59°39′50.0″N 10°45′34.9″E	Stagnosol	Dfb	6.4	7.4	876	603

^1^AGES, Austrian Agency for Health and Food Safety; AGS, Agroscope; BSA, Federal Plant Variety Office; CRAW, Walloon Agricultural Research Centre; ETH, Federal Institute of Technology; IRTA, Institute of Agrifood Research and Technology; LAMMC, Lithuanian Research Center for Agriculture and Forestry; NEBIH, National Food Chain Safety Office; NIBIO, Norwegian Institute of Bioeconomy Research; TUM, Technical University Munich; UKZUZ, Central Institute for Supervising and Testing in Agriculture.

^2^Soil type for all sites except NO-As according to World Reference Base (2022), NO-As according to World Reference Base (2014).

^3^Cfb, oceanic; Dfa, continental; Dfb, humid continental.

^4^MAP, mean annual precipitation (1991–2020); MAT, mean annual temperature (1991–2020).

^5^Data only available from 1 December 2022 to 31 August 2022.

**Table 2.** Site characteristics: management information for 11 sites in the growing season 2021–22.

Site abbreviation	Sowing density (grains m^−2^)	Sowing depth (cm)	Row width (cm)	Nitrogen fertilization (kg ha^−1^)^1^	Chemical plant protection	Growth regulators	Preceding crop	Irrigation (mm)
AT-Gn	300	3	12.5	130	Yes	No	Oil pumpkin	0
BE-Ge	275	2	15.6	150	No	Yes	Sugar beet	0
CH-Ca	350	2	15.5	130	Yes	Yes	Sunflower	0
CH-Es	400	2.5	12.5	130/85^1^	Yes	Yes	Winter wheat/grass-clover ley	0
CZ-Cr	350	4	12.5	130	Yes	No	*Phacelia*	0
DE-Fr	350	3	12.5	180	Yes	Yes	Winter wheat	0
DE-No	400	3	12	70	Yes	Yes	*Vicia sativa*	0
ES-Le^2^	450	2	15	0	Yes	No	Alfalfa	20 ^2^
HU-Sz	450	4.5	10.5	125	Yes	No	Maize	0
LT-Do	350	3	12.5	140	Yes	Yes	Winter wheat	0
NO-As	450	3	12.5	136.5	Yes	No	Unknown	0

^1^Nitrogen (N) fertilization was calculated according to mineral N content in soil determined in spring. In CH-Es, N fertilization differed between the two field replicates due to differences in pre-crops and soil mineral N contents.

^2^In Es-Le, the wheat received 20 mm of irrigation in spring in order to prevent drought-induced crop failure.

Daily climate data were gathered from nearby weather stations (AT-Gn (GeoSphere Austria, 2023), BE-Ge (RMI, 2023), CH-Ca (Federal Office of Meteorology and Climatology MeteoSwiss, 2022), CH-Es (Agrometeo, 2024), DE-Fr (Bayrisches Landesamt für Umwelt, 2023), DE-No (Agrarmeteorologisches Messnetz Sachsen, 2023), LT-Do (Lithuanian Hydrometeorological Service, 2022), CZ-Cr, ES-Le, HU-Sz and NO-As: data retrieved from on-site weather stations). Mean annual temperature and precipitation and mean temperature and sum of precipitation for the time period September 2021 to August 2022 were calculated for general site characterization (Table 1). Mean temperature and sum of precipitation for the time periods 1 week before sowing until harvest (Temp. season and Prec. season, respectively), 1 week before sowing until emergence (Temp. emergence and Prec. emergence, respectively), emergence until flowering (Temp. flowering and Prec. flowering, respectively), and flowering until harvest (Temp. harvest and Prec. harvest, respectively) were calculated for each site individually to characterize the specific weather conditions during the wheat growing season (Supplementary Data Table S1).

The ten winter wheat varieties chosen for the present study were commercially relevant in large parts of Europe and differed strongly in yield expectation based on their adaptability to certain environmental conditions. The included varieties were Altigo, Aurelius, Bernstein, Dagmar, Julie, Montalbano, MV Nador, Nogal, RGT Reform and Tenor (Table 3). At DE-Fr, the varieties Altigo and Tenor and at NO-As the varieties Aurelius and Tenor were not cultivated and therefore not included in our study for those sites.

**Table 3.** Ten winter wheat varieties used in this study, their abbreviation and year of release.

Variety	Variety abbreviation	Year of release^1^
Altigo	Al	2011
Aurelius	Au	2016
Bernstein	Be	2015
Dagmar	Da	2012
Julie	Ju	2014
Montalbano	Mo	2014
MV Nador	Na	2012
Nogal	No	2013
RGT Reform	Re	2014
Tenor	Te	2017

Once a variety has been registered in one EU country or in CH, it can be grown in any other EU country and in CH.

^1^Source: European plant variety protection EUPVP—Common Catalogue Information System (2023).

### Sampling, sample processing and measurements

#### Root and soil sampling

Root sampling was performed in July and August 2022. The sampling was explicitly conducted after harvest to simultaneously quantify root carbon inputs to soil as net root biomass, which was the main objective of the MaxRoot-C project (Heinemann *et al.*, 2025). At all sites except CH-Es, three field replicates per variety were sampled (30 experimental plots per site). In CH-Es, the ten varieties were part of a variety testing panel with over 100 varieties and two field replicates; two samples were taken from the first field replicate (serving as pseudo-replicates) and one from the second replicate.

Two well-established methods were used to quantify root traits (Gregory, 2006): (1) monolith excavation and (2) soil coring. (1) One 0.25 × 0.25 × 0.15 m (L × W × D) soil monolith per plot comprising two wheat rows was excavated, the entire soil volume was retrieved, and all crown roots were collected. (2) In addition, two soil cores per plot were taken with a soil auger (inner diameter 6 cm, outer diameter 8 cm), one directly on the crop row and one between crop rows, up to a depth of 1 m. The core was then retrieved from the rod and divided into five depth segments of 0.00–0.15, 0.15–0.30, 0.30–0.50, 0.50–0.75 and 0.75–1.00 m. The core samples from 0.00–0.15 m soil depth were not included in this study, as they contained large amounts of extraneous organic matter (roots from preceding crops, above-ground crop residues, organic amendments), which could not be differentiated from the recent wheat roots in a precise and efficient manner. From here on, the crown roots from the soil monoliths are referred to as topsoil roots (0.00–0.15 m soil depth) and the roots from the soil cores as subsoil roots (0.15–1.00 m soil depth).

Two extra soil cores were sampled from the centre of each field for the determination of soil characteristics. For the sampling period, all samples were stored at ambient temperature for a maximum of 10 d and thereafter cooled at 4 °C for a maximum of 60 d or frozen at −18 °C for a maximum of 12 months. Detailed information on sampling, sample processing, and measurements is given in Supplementary Data SIS1.

#### Root sample processing and measurements

Topsoil root samples were washed manually and subsoil root samples were washed using a root washing machine (Hydropneumatic Elutriation System; Gillison’s Variety Fabrication; Smucker *et al.*, 1982). The washed root samples were expelled into a 500-μm sieve, which might have underestimated root length by 20 % compared with a 250-μm sieve (Livesley *et al.*, 1999). Thereafter, the roots had to be further separated from remaining extraneous organic matter by hand. The subsoil root samples of ES-Le had exceptionally high proportions of roots of the preceding crop alfalfa (lucerne) in all soil depths and were therefore not subjected to root measurements by image analysis. Scanning was performed with an Epson Perfection V850 flatbed scanner with a custom-made Plexiglas^®^ tray (York, 2020). The subsoil roots were scanned in a water film, whereas the topsoil roots were scanned without water. To allow for easy 2-D scanning, two crown roots per sample were bisected and the four crown root halves were scanned (Supplementary Data Fig. S3).

#### Soil sample processing and measurements

Soil analyses were performed on 40 °C-dried and 2-mm-sieved samples. Water content, stone content (>2 mm) and bulk density were assessed by drying and weighing of the samples, particle size distribution (clay <2 μm, silt 2–50 μm, sand >50 μm) was measured with a robotic analyser (Skalar SP2000), soil pH was measured in 0.1 m CaCl_2_ solution at a ratio of 1:2.5, total carbon (C) and nitrogen (N) were measured by dry combustion (Leco TruMac CN Macro Determinator), inorganic C was determined by combusting aliquots for 16 h in a muffle furnace at 400 °C, and C and N were measured by elemental analysis. Organic C content was then calculated by subtracting the total inorganic C content from the total C content of these samples. Available soil phosphorus (P) was measured by Olsen extraction and colorimetry (Olsen, 1954). Data for clay content, bulk density, pH, total N, available P and total inorganic and organic C are presented in Supplementary Data Table S2.

#### Grain yield and gene sequencing

Grain yield was determined by the site managers and upscaled to Mg ha^−1^ at 15 % moisture (Table 4; more information in Heinemann *et al.* 2025). For genome sequencing, seeds of all ten varieties of three sites (LT-Do, CH-Es, ES-Le), a total of 30 samples, were provided by the site managers and stored in a cool dry place until further analysis. At SGS Institut Fresenius GmbH TraitGenetics Section, plants were grown from the seeds, the DNA was extracted from leaf material, and sequencing was performed with the Illumina Infinium 25 K array (Gogna *et al.*, 2022). Based on a cluster file developed for hexaploid wheat, the data were checked for quality and SGS provided a genotype table for the analysed samples.

**Table 4.** Mean, minimum and maximum grain yield (15 % moisture) of ten winter wheat varieties per site in Mg ha^−1^.

	Site										
	AT-Gn	BE-Ge	CH-Ca	CH-Es	CZ-Cr	DE-Fr	DE-No	ES-Le	HU-Sz	LT-Do	NO-As
Mean	10.2	7.9	3.5	8.7	9.8	6.52	9.3	4.2	3.6	7.6	6.2
Minimum	9.2	5.0	2.8	7.7	9.2	5.49	8.2	3.3	3.0	6.7	3.1
Maximum	11.2	9.8	4.1	9.8	10.5	7.54	10.3	4.9	3.9	8.4	7.6

Data are averaged across field replications and summarized across the ten varieties.

### Data analyses

#### Root image analysis

The images (Supplementary Data Fig. S3) were analysed using RhizoVision Explorer v2.0.3 (Seethepalli and York, 2021) and the algorithms described by Seethepalli *et al.* (2021). The specific settings are summarized in Supplementary Data Table S3. Although multiple root parameters were determined, we focused on root length and surface area for further analyses (Supplementary Data Table S4). In the diameter range over 4 mm of the fresh, bisected topsoil roots, which corresponded to the bisected part of the root crown adjoining the stem base, length and surface area were corrected for duplicate measurements.

#### Handling of missing data and upscaling

Of a total of 330 topsoil root (3 replicates × 10 varieties × 11 sites) and 2640 subsoil root (2 positions × 4 sampling depths × 3 replicates × 10 varieties × 11 sites) samples, 22 topsoil root and 340 subsoil root samples were missing. This concerned the data on the varieties Altigo and Tenor in DE-Fr and NO-As (not included in the panel), data for 0.15–1.00 m soil depth in ES-Le (contaminated samples), and data for 0.75–1.00 m soil depth and partly for 0.50–0.75 m soil depth in CH-Ca (limited sampling depth). These were replaced with N/A (not available) for data analyses.

The top- and subsoil root traits were upscaled following two different approaches. Topsoil root traits are reported per piece of crown root, while subsoil root traits are reported per area (m^2^ soil) (see Suitability of the study design in the Discussion section for a critical assessment of the two approaches). For the latter, data from soil cores sampled within and between wheat rows were combined using an approach that accounted for the spatial representativeness of the core positions. Given that row widths ranged between 10.5 and 15.6 cm across sites (Table 2), the relative proportions of root systems collected between wheat rows likely varied among sites. Root length and surface area of the subsoil roots were therefore upscaled to the soil surface area for each sampling position and depth segment individually and then summed over positions and depths (adapted from Frasier *et al.* (2016) and Hirte *et al.* (2021) for root biomass):


(1) $$root\;trait_{row\;upscaled}=\frac{root\;trait_{row}}{\pi *{\left(\frac{D}{2}\right)}^{2}}*\frac{D}{s}$$

(2) $$root\;trait_{inter-row\;upscaled}=\frac{root\;trait_{inter-row}}{\pi *{\left(\frac{D}{2}\right)}^{2}}*\frac{\left(s-D\right)}{s}$$

where *root trait_row upscaled_* and *root trait_inter-row upscaled_* are area-related root length (m root m^−2^ soil) or surface area (m^2^ root m^−2^ soil) within and between rows, respectively, *root trait_row_* and *root trait_inter-row_* are root length (m) or surface area (m^2^) per soil core within and between rows, respectively, *D* is the inner diameter of the sampling rod (m) and *s* is the distance between rows (m) (i.e. row width; Table 2). Upscaled subsoil root length and surface area per depth segment were obtained by summing the respective *root trait_row upscaled_* and *root trait_inter-row upscaled_*. In addition, the data for the individual depth segments were summed to two subsoil segments, 0.15–0.50 and 0.50–1.00 m. For data that were only available for either the row or the inter-row position, the data point for the missing position was estimated (details in Supplementary Data SIS2).

#### Statistical analysis of root data

First, general variability in non-transformed root trait data among varieties and sites was assessed by a random intercept model:


(3) $$\{\begin{array}{c} Y_{ijk}=\;\mu +\alpha_{i}+\;S_{j}+\;R_{k}+\;\varepsilon_{ijk}\\ \alpha_{i}∼N{\left(0,\sigma_{V}^{2}\right)}_{,}\;S_{i}∼N{\left(0,\sigma_{S}^{2}\right)}_{,}\;\;R_{k}∼N{\left(0,\sigma_{R}^{2}\right)}_{,\;\;}\varepsilon_{ijk}∼N(0,\sigma^{2}) \end{array}$$

where *Y_ijk_* is the value of the root trait (root length or surface area), *α_i_* is the random effect of variety *i*, *S_j_* is the random effect of site *j*, *R_k_* is the random effect of replicate *k*, and *ɛ_ijkl_* is the error term.

Second, differences in root length and surface area between varieties were evaluated by means of linear mixed effects models to account for the prominent hierarchical data structure (nested design) and different sources of variability. Prior to model fitting, root length and surface area were log-transformed to meet the assumption of homogeneous distribution of the residuals. Separate models were fitted to the data for the different aggregated soil layers:


(4) $$\{\begin{array}{c} \log\;(Y_{ijk})=\;\mu +\alpha_{i}+\;S_{j}+\;R_{k(j)}+\;\varepsilon_{ijk}\;\\ \;S_{i}∼N{\left(0,\sigma_{S}^{2}\right)}_{,}\;\;R_{k(j)}∼N{\left(0,\sigma_{R}^{2}\right)}_{,\;}\;\varepsilon_{ijk}∼N(0,\sigma^{2}) \end{array}$$

where *Y_ijk_* is the value of the root trait (root length or surface area), *α_i_* is the fixed effect of variety *i*, *S_j_* is the random effect of site *j*, *R_k(j)_* is the random effect of replicate *k* nested in site *j* and *ɛ_ijk_* is the error term.

Third, we tested the effects of the following pedoclimatic and management variables on root length and surface area using mixed effects models with an interaction term of variety and pedoclimatic or management variable (Supplementary Data Tables S1 and S2): Temp. season (°C), Temp. emergence (°C), Temp. flowering (°C), Temp. harvest (°C), Prec. season (mm), Prec. emergence (mm), Prec. flowering (mm), Prec. harvest (mm), Soil clay content (%), Soil BD (g cm^−3^), Soil pH, Soil N (%), Soil P (mg kg^−1^), Sowing density (grains m^−2^) and N fertilization (kg ha^−1^):


(5) $$\{\begin{array}{c} \log\;(Y_{ijkl})=\;\mu +\alpha_{i}+\beta_{j}+{\left(\alpha \beta \right)}_{ij}\\ \;\;\;+\;S_{k}+\;R_{l(k)}+\;\varepsilon_{ijkl}\;\\ \;S_{k}∼N{\left(0,\sigma_{S}^{2}\right)}_{,}\;\;R_{l(k)}∼N{\left(0,\sigma_{R}^{2}\right)}_{,\;}\;\varepsilon_{ijkl}∼N(0,\sigma^{2}) \end{array}$$

where *Y_ijk_* is the value of the root trait (root length or surface area), *α_i_* is the fixed effect of variety *i*, *β_j_* is the fixed effect of pedoclimatic variable *j*, (*αβ*)*_ij_* is the fixed interaction effect, *S_k_* is the random effect of site *k*, *R_l_*_(*k*)_ is the random effect of replicate *l* nested in site *k* and *ɛ_ijkl_* is the error term. A multivariate Pearson correlation analysis revealed several prominent correlations between the pedoclimatic and management variables, most importantly between the climate variables (Supplementary Data Fig. S4).

The relative importance of all variables for root length and surface area was assessed using a random forest model (details in Supplementary Data SIS2).

Fourth, the relationships between root length and surface area and yield of individual varieties were tested using a heteroscedastic mixed effects model (Addy *et al.*, 2022) with an interaction term of variety and root length or surface area:


(6) $$\{\begin{array}{c} Z_{ijkl}=\;\mu +\alpha_{i}+\;\log\;(Y_{j})+{\left(\alpha log(Y)\right)}_{ij}\\ \;\;\;\;\;\;\;\;+\;S_{k}+\;R_{l(k)}+\;\varepsilon_{ijkl}\;\\ \;S_{k}∼N{\left(0,\sigma_{S}^{2}\right)}_{,}\;\;R_{l(k)}∼N{\left(0,\sigma_{R}^{2}\right)}_{,\;}\;\varepsilon_{ijkl}∼N(0,\sigma_{i}^{2}) \end{array}$$

where *Z_ijkl_* is the yield, *α_i_* is the fixed effect of variety *i*, log(*Y*)*j* is the fixed effect of root trait *j*, (*α*log(*Y*))*_ij_* is the fixed interaction effect, *S_k_* is the random effect of site *k*, *R_l_*_(*k*)_ is the random effect of replicate *l* nested in site *k* and *ɛ_ijkl_* is the error term. edna The variance function structure grouped by variety allowed for handling the significant heterogeneity of variance in the residuals (Pinheiro and Bates, 2006).

Fifth, the relationships between root length and surface area and yield of individual varieties under varying pedoclimatic conditions were tested using a heteroscedastic mixed effects model with a three-way interaction term of variety, root length or surface area, and pedoclimatic variable:


(7) $$\{\begin{array}{c} Z_{ijklm}=\;\mu +\alpha_{i}+\;\beta_{j}+\;\log\;(Y_{k})+\;{\left(\alpha \beta \right)}_{ij}+{\left(\alpha \log\;(Y)\right)}_{ik}\\ \;\;\;\;\;\;\;\;\;\;\;\;\;\;\;+\;{\left(\beta \log\;(Y)\right)}_{\;jk}+\;{\left(\alpha \beta \log\;(Y)\right)}_{ijk}\\ \;\;\;\;\;\;\;\;\;\;\;\;\;\;\;\;\;\;\;\;\;\;\;\;\;\;\;\;\;\;\;\;\;\;\;\;\;\;\;+\;S_{l}+\;R_{m(l)}+\;\varepsilon_{ijklm}\;\\ \;S_{l}∼N{\left(0,\sigma_{S}^{2}\right)}_{,}\;\;R_{m(l)}∼N{\left(0,\sigma_{R}^{2}\right)}_{,\;}\;\varepsilon_{ijklm}∼N(0,\sigma_{i}^{2}) \end{array}$$

where *Z_ijkl_* is the yield, *α_i_* is the fixed effect of variety *i*, *β_j_* is the fixed effect of pedoclimatic variable *j*, log(*Y*)*_k_* is the fixed effect of root trait *k*, (*αβ*)*_ij_*, (*α* log(*Y*))*_ik_*, (*β* log(*Y*))*_jk_* and (*αβ* log(*Y*))*_ijk_* are the fixed two- and three-way interaction effects, *S_l_* is the random effect of site *l*, *R_m_*_(*l*)_ is the random effect of replicate *m* nested in site *l* and *ɛ_ijklm_* is the error term.

For models 4–7, outliers were excluded on the basis of the 95 % confidence interval of the standardized residuals in all final models. The models were checked for influential cases by computing Cook’s distance and for heteroscedasticity by performing Levene’s test. Overall model performances were checked by pseudo-*R*^2^ (marginal and conditional *R*^2^) for generalized mixed models and the normality of the residuals (QQ plots). For all models, details on model diagnostics are given in Supplementary Data SIS2.

Differences in root length or surface area between varieties (model 4) were tested by ANOVA and subsequent multiple pairwise comparison of estimated marginal means. Differences in slopes for the pedoclimatic and management variables and root length or surface area and yield between varieties (models 5 and 6) were tested by multiple pairwise comparisons of estimated marginal trends. Changes in slope between root length or surface area and yield with changing pedoclimatic conditions for every variety were tested by multiple pairwise comparisons between the slopes of the mean pedoclimatic value ± 1 standard deviation (Aiken, 1991). For all multiple comparisons, Sidak adjustment of *P*-values was applied and a significance level of *α* = 0.05 was used.

Finally, we performed a broad-sense heritability analysis (*H*^2^_piepho_) on root length and surface area based on a mixed model approach to account for the unbalanced trial design (Piepho and Möhring, 2007). Best linear unbiased estimators (BLUEs) were used for fixed effects and best linear unbiased predictors (BLUPs) for random effects:


(8) $$Fixed\;model=\{\begin{array}{c} Y_{ijk}=\;\mu +\;\alpha_{i}+\;S_{j}+\;{\left(\alpha S\right)}_{ij}+\;\varepsilon_{ijk}\;\\ \;S_{i}∼N{\left(0,\sigma_{S}^{2}\right)}_{,}\;\;{\left(\alpha S\right)}_{ij}∼N{\left(0,\sigma_{VS}^{2}\right)}_{,\;}\;\varepsilon_{ijk}∼N(0,\sigma^{2}) \end{array}$$

(9) $$Random\;model\;=\;\{\begin{array}{c} Y_{ijk}=\;\mu +\;\alpha_{i}\;+\;S_{j}\;+{\left(\alpha S\right)}_{ij}+\;\varepsilon_{ijk}\;\\ \;S_{i}∼N{\left(0,\sigma_{S}^{2}\right)}_{,}\;\;\alpha_{i}∼N{\left(0,\sigma_{V}^{2}\right)}_{,\;}\\ {\left(\alpha S\right)}_{ij}∼N{\left(0,\sigma_{VS}^{2}\right)}_{,\;}\;\varepsilon_{ijk}∼N(0,\sigma^{2}) \end{array}$$

where *α_i_* is the fixed effect of variety *i* in the fixed model and the random effect of variety *i* in the random model, *S_j_* is the random effect of site *j*, (*αS*)*_ij_* is the random interaction effect and *ɛ_ijk_* is the error term. Heritability was then calculated according to Piepho and Möhring (2007):


(10) $$H_{\;piepho.}^{2}=\;\frac{\sigma_{g}^{2}}{\sigma_{g}^{2}+\frac{\bar{v}}{2}}$$

where *σ*^2^*_g_* is the variance of a genotype calculated in formula (8) and $\bar{v}$ is the mean variance of a difference of two adjusted environment means (BLUE) calculated in formula (9).

#### Genetics

We prepared the genotype table by translating it from IUPAC–IUB (International Union of Pure and Applied Chemistry–International Union of Biochemistry) code to four bases (A, T, C, G) and determined the major and minor allele for every single-nucleotide polymorphism (SNP) by determining respectively the more and less frequently occurring allele in the sample population. The minor allele was coded as 1 and the major allele as 0 (Gauch *et al.*, 2019). The genotype table was further filtered to contain only SNPs with complete observations and was subsequently double-centred (Gauch *et al.*, 2019). Further, we performed double-centred principal component analysis (DC-PCA) and agglomerative hierarchical *k*-means cluster analysis to estimate the genetic (dis)similarity among the varieties. For these analyses, only SNPs that varied within the individuals (non-monomorphic) and with no missing observations were used.

#### Software

All analyses were performed in the R environment, version 4.2.3 (R Core Team, 2023), with the R packages tidyverse (Wickham *et al.*, 2019), readxl (Wickham and Bryan, 2023), reshape (Wickham, 2007) and reshape2 (Wickham, 2007) for data management, car (Fox and Weisberg, 2019), grafify (Shenoy, 2021), emmeans (functions emmeans and emtrends) (Lenth, 2023), multcomp (function cld) (Hothorn *et al.*, 2008), MuMIn (function r.squaredGLMM) (Bartoń, 2023) and predictmeans (function residplot) (Luo *et al.*, 2022) for statistical analysis, DALEX (function explain) (Biecek, 2018), inti (function H2cal) (Lozano-Isla, 2023), nlme (function lme) (Pinheiro *et al.*, 2023), lme4 (function lmer) (Bates *et al.*, 2015) and randomForest (function randomForest) (Liaw and Wiener, 2002) for model fitting, MLMOI (function moimport) (Hashemi and Schneider, 2020) for genotype data translation, and ggplot2 (Wickham, 2016), ggbiplot (Vu, 2011), ggpubr (Kassambara, 2023), scales (Wickham and Seidel, 2022), sjmisc (Lüdeke, 2018) and sjPlot (Lüdeke, 2023) for visualization.

## RESULTS

### Genotypic variation in root length and surface area

The topsoil roots had on average a root length of 1.4 m root piece^−1^ and a root surface area of 0.039 m^2^ root piece^−1^ across varieties and sites. At 0.15–0.50 and 0.50–1.00 m soil depth, respectively, root length averaged 5007 and 5300 m root m^−2^ soil and root surface area averaged 40 and 43 m^2^ root m^−2^ soil across varieties and sites. The variability in the data was ∼5–15 times higher for the sites than for the varieties (Table 5). The residual standard deviation was about one-third of the total standard deviation, indicating that the chosen models covered the sources of variance to a major part (Table 5).

**Table 5.** Summary statistics on root length and surface area in the topsoil (0.00–0.15 m, per piece) and subsoil (0.15–0.50 and 0.50–1.00 m, per area) of ten winter wheat varieties at 11 sites in Europe.

	Topsoil roots		Subsoil roots at 0.15–0.50 m		Subsoil roots at 0.50–1.00 m	
	Length (m piece^−1^)	Surface area (m^2^ piece^−1^)	Length (m root m^−2^ soil)	Surface area (m^2^ root m^−2^ soil)	Length (m root m^−2^ soil)	Surface area (m^2^ root m^−2^ soil)
Summary						
Mean^1^	1.4	0.039	5007	40	5300	43
Median^1^	1.4	0.040	5031	39	5387	44
Min	0.4	0.011	1448	13	407	5
Max	2.7	0.089	8841	80	14′923	96
Standard deviation of random effects						
Standard deviation^1^	0.1	0.004	384	4	402	5
Variety	0.1	0.003	198	2.5	236	3.5
Site	0.5	0.015	1720	14.5	3740	25.8
Replicate^2^	N/A	N/A	137	0.6	615	4.3
Residual	0.4	0.013	1451	12.5	1905	16.6
Significant differences						
Variety	***	***	n.s.	*	*	*
*R*^2^ marginal	0.035	0.040	0.019	0.026	0.016	0.022
*R*^2^ conditional	0.72	0.72	0.70	0.69	0.82	0.76
Heritability						
*H*^2^	0.56	0.63	0.41	0.57	0.59	0.62

Standard deviations of random effects were derived from a random intercept model with linear combinations of variety and site as random effects. Differences in root traits among varieties were derived from a mixed effects model with variety as fixed effect and site and replicate as nested random effects. The goodness of model fit is indicated by the marginal *R*^2^ for the fixed effects and the conditional *R*^2^ for the whole model. Indication of significance level for differences among varieties based on ANOVA are represented by asterisks (*P < 0.05, **P < 0.01, ***P < 0.001, n.s. not significant).

^1^Across field replications and varieties.

^2^Random intercept model including replicate showed a singular fit for topsoil root traits.

**Fig. 1.**
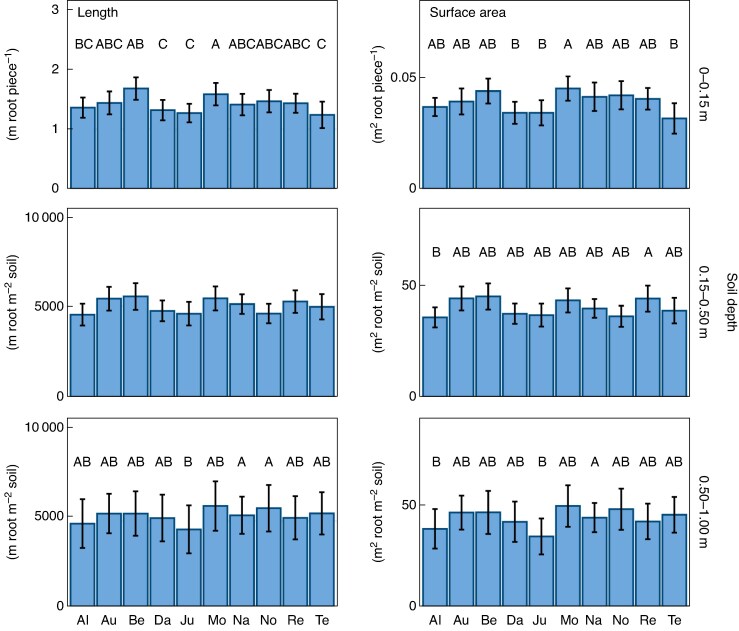
Root length and surface area at three soil depths (depth 0.00–0.15 m, per piece; depth 0.15–0.50 m and 0.50–1.00 m, per area) of ten winter wheat varieties at 11 sites in Europe. Bars depict mean values of 10 or 11 sites averaged over three replications and error bars depict standard errors of 10 or 11 sites. Groupings according to the outcome of the statistical analysis are indicated by letters above the columns (significant difference if not sharing a letter; comparison of varieties in individual soil depths). Missing letters indicate non-significant differences. Please refer to Table 3 for variety abbreviations.

The varieties exhibited almost a 2-fold variation in root length and surface area within sites. The topsoil roots ranged between 1.2 and 1.7 m root piece^−1^ in root length and 0.031 and 0.045 m^2^ root piece^−1^ in root surface area among varieties and differed significantly in both traits (Fig. 1). Montalbano consistently showed the highest root length and surface area, along with Bernstein, RGT Reform and Aurelius (Fig. 1). In contrast Julie, Dagmar and Tenor exhibited the lowest values for both root traits. At a soil depth of 0.15–0.50 m, the root length did not significantly differ among the varieties (average 5007 m root m^−2^ soil), but root surface area was highest for RGT Reform (45 m^2^ root m^−2^ soil), lowest for Altigo (36 m^2^ root m^−2^ soil) and intermediate for all other varieties (Table 5; Fig. 1). At a soil depth of 0.50–1.00 m, both root traits varied significantly among the varieties, ranging from 4508 to 5877 m root m^−2^ soil in root length and 35 to 49 m^2^ root m^−2^ soil in root surface area. MV Nador and Nogal had the highest values, while Julie had the lowest values for both traits (Table 5; Fig. 1). Heritability ranged between 0.56 and 0.63 for both root traits in all soil depths except for root length at 0.15–0.50 m depth (*H*^2^ = 0.41) (Table 5).

### Pedoclimatic and management effects on root length and surface area

The main effects of the pedoclimatic and management variables were significant for the root traits in the subsoil only (Table 6). Irrespective of variety, both root length and surface area were positively related to different temperature variables and root surface area was also negatively related to precipitation over the entire season. At 0.50–1.00 m soil depth only, root length and surface area were negatively related to soil bulk density and root surface area was also negatively related to N fertilization. The multivariate random forest model analysis revealed temperature, precipitation, soil clay content and soil bulk density as the most important variables for root length and surface area in the topsoil, while temperature, precipitation and soil bulk density were the most important variables at 0.15–0.50 m soil depth, and temperature, soil bulk density and N fertilization at 0.50–1.00 m soil depth (Supplementary Data Figs S5 and S6).

**Table 6.** Significance of correlation coefficients for main effects of pedoclimatic and management variables for (log-transformed) root length and surface area based on linear mixed models. Please refer to Sites and wheat varieties in the Materials and methods section for variable abbreviations.

Variable	Soil depth					
	0.00–0.15 m		0.15–0.50 m		0.50–1.00 m	
	Length	Surface area	Length	Surface area	Length	Surface area
Temp. season (°C)	n.s.	n.s.	0.059	0.073	0.069	**0.046 (+)**
Temp. emergence (°C)	n.s.	n.s.	n.s.	n.s.	n.s.	n.s.
Temp. flowering (°C)	n.s.	n.s.	**0.036 (+)**	0.095	**0.039 (+)**	**0.031 (+)**
Temp. harvest (°C)	n.s.	n.s.	n.s.	n.s.	**0.002 (+)**	**0.008 (+)**
Prec. season (mm)	n.s.	n.s.	0.081	**0.026 (−)**	0.069	**0.024 (−)**
Prec. emergence (mm)	n.s.	n.s.	n.s.	n.s.	n.s.	0.081
Prec. flowering (mm)	n.s.	n.s.	n.s.	0.086	n.s.	n.s.
Prec. harvest (mm)	n.s.	n.s.	n.s.	n.s.	n.s.	n.s.
Soil clay content (%)	n.s.	n.s.	n.s.	n.s.	0.095	n.s.
Soil bulk density (g cm^−3^)	n.s.	n.s.	n.s.	n.s.	**0.004 (−)**	**0.002 (−)**
Soil pH	n.s.	n.s.	n.s.	n.s.	n.s.	0.056
Soil N (%)	n.s.	n.s.	n.s.	n.s.	n.s.	n.s.
Soil P (mg kg^−1^)	n.s.	n.s.	n.s.	n.s.	n.s.	n.s.
Sowing density (grains m^−2^)	n.s.	n.s.	n.s.	n.s.	n.s.	n.s.
N fertilization (kg ha^−1^)	n.s.	n.s.	n.s.	n.s.	n.s.	**0.049 (−)**

*P* values <0.05 are highlighted in bold font and *P* values >0.1 are shown as n.s. The direction of significant effects is indicated in brackets: +, positive; −, negative.

For both root traits and all soil depths, significant interactions between variety and pedoclimatic and management variables were observed in multiple instances. However, after Sidak *P*-value adjustment for multiple pairwise comparison, only a few varieties showed slopes significantly different from zero and from each other. Soil and climate variables, rather than management, were significant drivers of root length and surface area of some varieties (Supplementary Data Figs S7–S9). In the topsoil, the root traits were significantly related to soil clay content (negative), soil bulk density (positive), soil pH (negative), temperature before emergence (positive) and temperature before harvest (negative). These effects were most prominent in the varieties Nogal and Aurelius (Supplementary Data Fig. S7). In contrast, all varieties showed significant correlations with one or more pedoclimatic variables in their subsoil root traits, with some varieties responding more strongly than others. An increase in soil pH and temperature before flowering, before harvest, and over the season, as well as a decrease in precipitation before emergence and over the season was correlated with an increase in both root traits at 0.15–0.50 and/or 0.50–1.00 m soil depth (Supplementary Data Figs S8 and S9).

### Variety-specific relationships between root length and surface area and yield

**Fig. 2.**
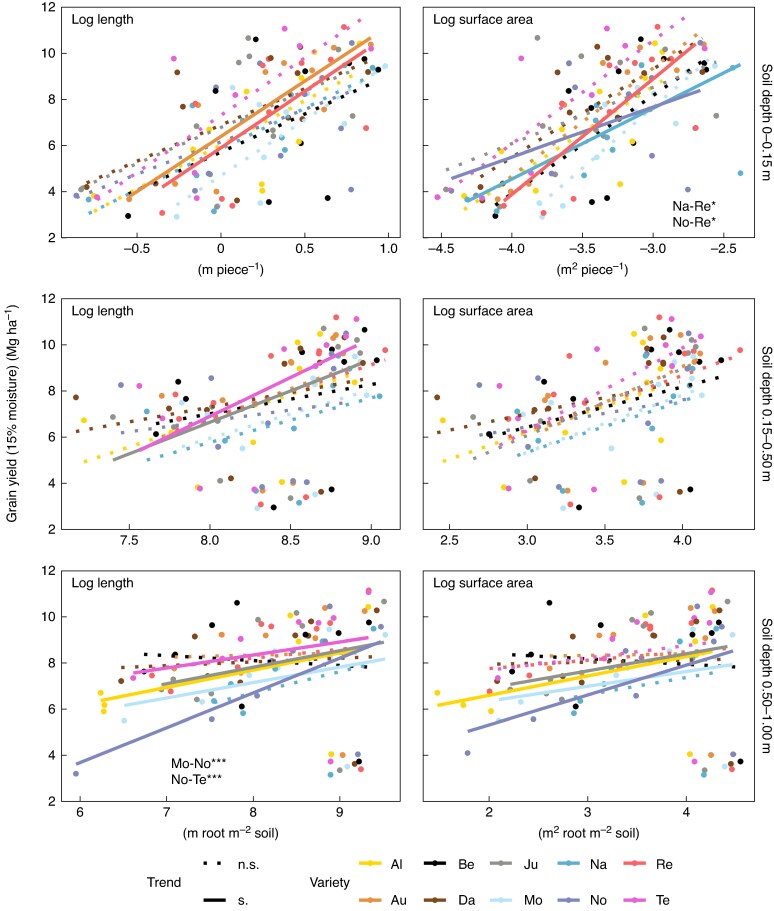
Grain yield of ten winter wheat varieties at 11 sites in Europe as related to root length and surface area of the topsoil and subsoil roots. Points are average values per variety and site. Significance of trend line is derived from mixed model output with site and replicate as nested random effects. Solid lines indicate a trend significantly different from zero. Slopes differing significantly from each other are indicated in the bottom right of the respective panel. Indication of significance level for differences among slopes based on multiple pairwise comparison and Sidak adjustment of *P*-values is represented by asterisks (*P < 0.05, **P < 0.01, ***P < 0.001). Trend: n.s., not significant; s., significant. Please refer to Table 3 for variety abbreviations.

Yields of several varieties across sites were significantly positively correlated with longer roots and greater surface area (Fig. 2). The yields of varieties Aurelius, MV Nador and RGT Reform showed significant relationships with root length or surface area in the topsoil only, whereas the yields of varieties Altigo, Julie, Montalbano and Tenor showed significant relationships with root length or surface area in the subsoil only. Nogal was the only variety with significant relationships of both topsoil and subsoil root traits with yield, while Bernstein and Dagmar did not show any significant relationships between yield and root traits.

**Fig. 3.**
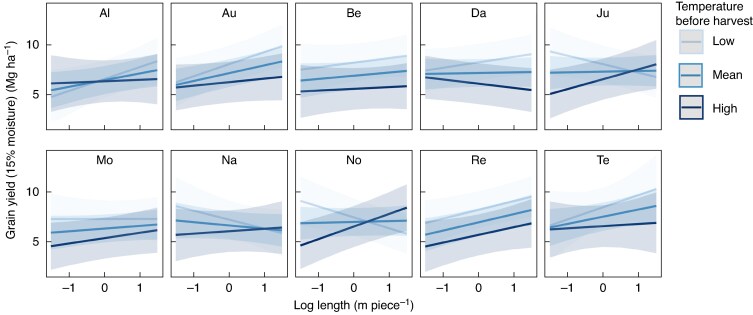
Relationship between root length in the topsoil (0.00–0.15 m soil depth) and predicted yield of ten winter wheat varieties at different values of temperature before harvest. Low, mean and high scenarios indicate mean − 1 s.d. (18.0 °C), mean (19.6 °C) and mean + 1 s.d. (21.3 °C) temperature, respectively. Please refer to Table 3 for variety abbreviations.

**Fig. 4.**
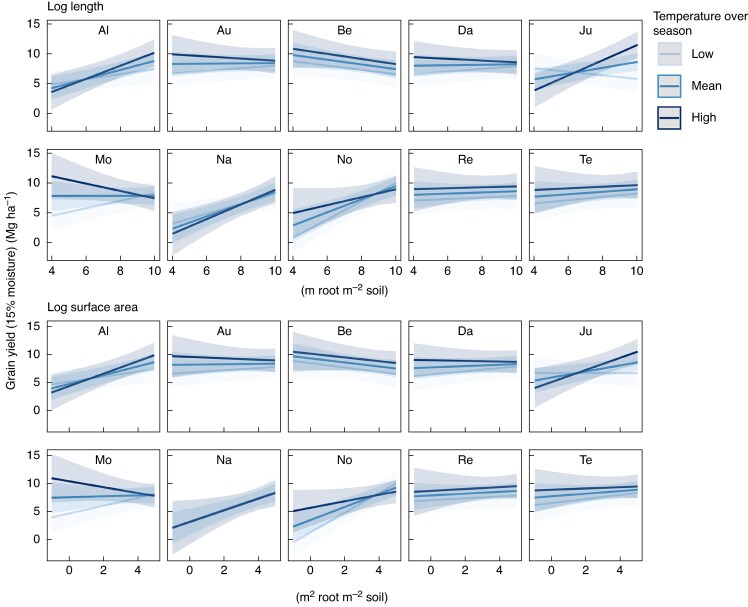
Relationship between root length and surface area at 0.50–1.00 m soil depth and predicted yield of ten winter wheat varieties at different values of temperature over the season. Low, mean and high scenarios indicate mean − 1 s.d. (7.6 °C), mean (8.6 °C) and mean + 1 s.d. (9.6 °C) temperature, respectively. Please refer to Table 3 for variety abbreviations.

**Fig. 5.**
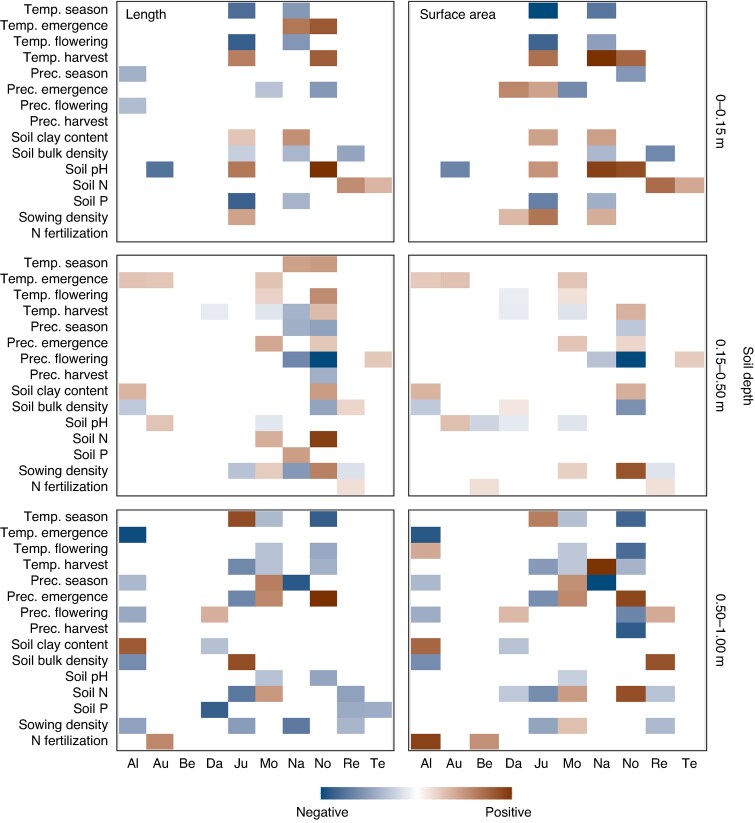
Indication of significant slope differences of the relationship between yield and root length and surface area of ten winter wheat varieties between the low (mean – 1 s.d.) and high (mean + 1 s.d.) scenario of a pedoclimatic or management variable. A blue tile indicates a negative difference in slopes and a red tile indicates a positive difference, i.e. the relationship between yield and root length or surface area becomes less and more pronounced, respectively, with increasing values of the pedoclimatic or management variables. Please refer to Table 3 for variety abbreviations and to Sites and wheat varieties in the Materials and methods section for variable abbreviations.

For all varieties, the relationship between yield and root length and surface area varied significantly with changing pedoclimatic and management conditions, but to very different extents (Figs 3–5). For instance, varieties Julie, MV Nador and Nogal showed a strong positive relationship between grain yield and topsoil root length at high temperatures before harvest, whereas this relationship turned negative at low temperatures before harvest (Fig. 3). For Montalbano, the relationship between yield and root length and surface area at 0.50–1.00 m soil depth reversed from positive to negative with increasing temperatures over the season (Fig. 4). For Altigo, the positive relationship of root length and surface area to yield became significantly steeper as temperatures increased (Fig. 4).

There were notable differences in the interaction between variety and pedoclimatic or management variables between topsoil and subsoil root traits (Fig. 5). In the topsoil, Julie and MV Nador showed the most prominent differences in the relationship between yield and root length and surface area under varying pedoclimatic conditions. In contrast, in the subsoil, Altigo, Julie, Montalbano (0.50–1.00 m soil depth only) and Nogal (0.15–0.50 and 0.50–1.00 m soil depth) showed the most prominent differences, both in terms of number of significant interactions and effect sizes. For the other varieties, changes in the relationship between yield and root traits under changing pedoclimatic and management conditions were generally less pronounced, irrespective of soil depth.

### Genetic similarities of wheat varieties

More than 50 % of the variance in the SNP data was explained by the first four interaction principal components (IPCs) of the DC-PCA analysis, with a clear decrease for the tenth component (Supplementary Data Fig. S10). Two varieties, MV Nador and Nogal, were prominently separated from the other varieties in the first IPC (17 % explained variance) and from each other in the second IPC (14 % explained variance). In the third IPC, varieties Bernstein and Dagmar represented the extremes at both ends and in the fourth IPC varieties Altigo and Julie differed the most from the other varieties (Supplementary Data Fig. S10). A similar clustering of genotypes was revealed by agglomerative hierarchical *k*-means cluster analysis, where MV Nador and Nogal, Dagmar and Tenor, Bernstein and Montalbano, and Altigo, Julie, RGT Reform and Aurelius formed the most prominent clusters (Supplementary Data Fig. S11).

## DISCUSSION

### Genotypic and environmental influences on root trait variability

Both root length and surface area varied strongly, and this variation was about 10 times greater among sites than varieties. In spite of the dominant site effect, we also observed a clear genotypic pattern in root length and surface area irrespective of site. Bernstein and Montalbano produced longer roots with a larger surface area in the topsoil, whereas MV Nador and Nogal had greater root length and larger root surface area in deeper soil layers. Although publicly available pedigree information does not indicate any shared breeding history among these ten varieties, our genetic analysis reveals a higher degree of relatedness between MV Nador and Nogal, as well as between Bernstein and Montalbano, than between other pairs of the ten varieties. This is represented by the proximity of their IPC scores in the DC-PCA and the short vertical distances in the cluster analysis dendrogram (Zhang *et al*., 2017). Moreover, considering that MV Nador (country of origin: Hungary) and Nogal (France; registered in Spain) are cultivated more frequently in Southern Europe, while Bernstein (Germany) and Montalbano (Switzerland) are typical of Central European regions, it is plausible that genetically driven differences in rooting patterns may reflect environmental adaptation. However, further research would be needed to confirm such associations.

The heritability for root length from 0.41 to 0.59 is in line with previous studies, which reported values of 0.62 in soil (Monyo and Whittington, 1970) and 0.48–0.70 in hydroponic culture (Xu *et al.*, 2021). High values for traits differing between varieties can be attributed to a significant genetic influence on the phenotypic variance of these traits (Piepho and Möhring, 2007). Conversely, low values for non-significant traits do not necessarily exclude genetic influence but may result from small mean differences and/or large error variances in the phenotype, often caused by high environmental influence (Oldenbroek and van der Waaij, 2015). Heritability for root surface area was generally higher (0.57–0.63) than that for root length, which supports the outcomes of the mixed model approach. In other studies, heritability estimates varied for root traits due to varying stages of growth and environments where genotypes were assessed (X. Guo *et al.*, 2020; H. Guo *et al.*, 2021 ; Timaeus *et al.*, 2021). The variation in heritability values suggests that genotypic expression is influenced by many genes and affected by environmental factors and their interactions (Mathew and Shimelis, 2022).

The significant variation in root traits among sites and the identified genotypic patterns provide deeper insights into how different wheat varieties adapt to varying environmental conditions. The discovery of genotypic subgroups with distinct root traits, such as those favouring topsoil exploration or deeper root proliferation, allows breeders to target specific traits for improvement. For instance, varieties like MV Nador and Nogal, which seem to be adapted to warm and dry environments with deeper roots, could be further used in new crosses for regions experiencing similar conditions.

### Pedoclimatic effects on root length and surface area

Temperature between emergence and harvest was a major driver of both root length and surface area. In the subsoil, all varieties showed higher root length and surface area with higher temperature, whereas in the topsoil only individual varieties, most prominently Nogal and Aurelius, were affected. During the 2021–22 wheat growing season, the natural climate gradient across Europe was intensified, with greater temperature and precipitation anomalies in the south compared with the north (Tripathy and Mishra, 2023). Except for DE-Fr, HU-Sz and LT-Do, temperatures in 2021–22 were 1–2.5 °C above MAT, and precipitation was on average 30 % lower than MAP at all sites. With the lack of rain and higher than normal temperatures, it is likely that evaporation also increased (Solomon *et al.*, 2007), potentially inducing drought stress at several sites.

Our data suggest that an overall warmer climate stimulates root growth in deeper soil layers irrespective of variety. Plants have an optimal temperature range for root growth and functioning, which ranges between 14 and 18 °C for wheat roots (Porter and Gawith, 1999). In warmer areas, increased temperatures can reduce root development in warmer topsoil layers while promoting root development in the cooler subsoil layers (Ribeiro *et al.*, 2014; Koevoets *et al.*, 2016; Calleja Cabrera *et al.*, 2020). High temperatures affect cell division and differentiation, reducing plant growth and development (Qi and Zhang, 2020; Liu *et al.*, 2022), and alter the stability of membranes, proteins, nucleic acids and cytoskeleton components (Vu *et al.*, 2019). In contrast, deeper soil layers often provide more favourable conditions for root growth due to lower and more stable temperatures resulting from the natural soil temperature gradient (Lynch and Wojciechowski, 2015).

Additionally, higher temperatures lead to increased evapotranspiration (Goyal, 2004; Solomon *et al.*, 2007), increasing water uptake from deeper soil layers (Asseng *et al.*, 1998). Deep roots have been considered to be one of the most effective ways to facilitate full utilization of subsoil water when topsoil water is not available under drought conditions (Lopes and Reynolds, 2010; Gowda *et al.*, 2011; Lynch, 2018; Maqbool *et al.*, 2022; Shoaib *et al.*, 2022). This is reflected in the data presented herein, which showed increases in root surface area in deep soil with decreasing precipitation over the entire season. During drought, plants reduce water use by closing stomata, which lowers CO_2_ intake and photosynthesis, ultimately reducing biomass production (Chaves *et al.*, 2002). While root growth initially slows, drought avoidance mechanisms soon promote increased primary and secondary root growth, extending into deeper or moister soil layers (Dinneny, 2019). This expansion of root surface area enhances water uptake under limited moisture conditions.

Besides temperature, soil bulk density and N fertilization were universal drivers of root length and surface area at a soil depth of 0.50–1.00 m, irrespective of variety. Generally, more compacted soil leads to reduced root length, surface area and dry matter, but may lead to a larger root diameter (Merotto and Mundstock, 1999; Rich and Watt, 2013), which is consistent with our findings for root length and surface area. Higher bulk density increases mechanical resistance, requiring more energy for root penetration and reducing root elongation (Bengough *et al.*, 2011; Kolb *et al.*, 2017). Compacted soils also have reduced porosity, which limits water infiltration, aeration and nutrient diffusion (Lipiec *et al.*, 2012). As a result, plants reduce root proliferation in these zones due to poor resource availability (Whalley *et al.*, 2005).

Being highly mobile in soils, N is generally the most limiting nutrient in arable farming, together with P (Koevoets *et al.*, 2016). The growth of plant roots is locally stimulated by N uptake, although, in total, less photosynthates are allocated to root growth as N availability increases (Rasse, 2002). Hence, low N fertilization appears to stimulate deep root growth as a result of acquiring proportionally more N from deeper soil (Rasse, 2002; Koevoets *et al.*, 2016). This supports previous findings where topsoil root biomass of winter wheat was negatively correlated with N fertilization, while subsoil root biomass was positively correlated with precipitation under wet spring conditions, suggesting that root production followed the leaching of N (Hirte *et al.*, 2018). The universal importance of N for root traits in the subsoil suggests that root foraging for this nutrient is largely independent of climate conditions.

### Interrelation of root plasticity and grain yield under varying pedoclimatic conditions

Grain yield was positively related to an increase in both root length and surface area but whether this link was related to the top- or subsoil was highly dependent on the variety. Among the ten varieties included in our study, three showed a distinct relationship of yield to topsoil root traits, four to subsoil root traits, one to both and two to neither. Several studies have suggested a positive effect of more and deeper roots on grain yield, particularly through increased water and nutrient uptake and consequently higher drought adaptation, which are beneficial for crop yields (Kirkegaard *et al.*, 2007; Maqbool *et al.*, 2022; Odone *et al.*, 2023). Deeper roots are also associated with cooler crop canopies and are correlated with more root biomass, both potentially increasing crop yield (Lopes and Reynolds, 2010; Li *et al.*, 2019; Heinemann *et al.*, 2023, 2025). These benefits of increased root length in deep soil appear to come at no ‘cost’ to shoot growth or yield and as such should remain a target for breeding (Severini *et al.*, 2020). This encourages the prospect of successfully selecting wheat varieties with an improved root system to achieve higher yields in warmer environments.

Under changing environmental conditions, the relationship between root traits and grain yield changed significantly for some varieties. Across sites, Altigo, Julie, Montalbano, MV Nador and Nogal demonstrated high root plasticity and the potential to sustain yields under increasingly harsh climatic conditions. This was particularly prominent for Altigo and Julie, which displayed increased deep root length and surface area with rising temperatures and at the same time proportionally higher yield increases. For Nogal, the positive relationship between increased deep root length and surface area and yield was more pronounced at the cooler than the warmer sites. By contrast, Montalbano only showed yield increases with increased deep root length and surface area at low temperatures but yield decreases at high temperatures. The importance of root plasticity for sustaining yields under variable conditions has also been proposed for rice under drought and nutrient stresses (Sandhu *et al.*, 2016; Xie *et al.*, 2021), for wheat under soil compaction (Correa *et al.*, 2019) and for maize under different water and nutrient scenarios (Hochholdinger and Tuberosa, 2009). Overall, root phenotypic plasticity has been proposed as a breeding target for developing more productive crops in variable environments, such as cool, temperate and warm climates (Schneider and Lynch, 2020).

For the varieties without a notable relationship between changes in yield and changes in root length or surface area, physiological processes or root traits not measured in our study might be more important for yield formation than the included traits. Among others, seedling root growth supports early soil exploration, promoting better nutrient and water uptake during crop establishment (Xie *et al.*, 2017). Root oxidation activity enhances rhizosphere conditions by releasing oxygen, aiding nutrient uptake by roots in waterlogged or anaerobic soils (Yang *et al.*, 2012). A steeper root growth angle promotes deeper rooting, increasing access to subsoil moisture and improving drought resilience and yield stability (Lynch, 2013). These traits or processes also contribute to yield formation, though their relative significance may vary depending on the variety.

In summary, the ten varieties can be broadly grouped according to distinct patterns of interrelationships between root plasticity and yield: a consistent relationship where changes in yield are proportional to changes in root traits irrespective of environmental conditions and a varying relationship where changes in yield become more or less pronounced with changes in root traits depending on the environmental conditions. These strategies might have different advantages in different environments: when the availability of soil resources such as water or nutrients varies frequently, varieties may benefit more from a consistent relationship to ensure a positive yield return on the investment in metabolite allocation to roots. By contrast, when the availability of resources remains stable, irrespective of sufficiency or deficiency for optimal yield, varieties might keep their metabolic costs to a minimum when a positive yield return on the investment in metabolite allocation to roots only occurs under these particular growth conditions. Among the ten varieties, some exhibit both consistent and variable relationships between yield and root length or surface area, particularly in the subsoil, indicating partial agreement rather than a clear separation. Hence, breeding efforts could simultaneously pursue both strategies, leading to varieties suited for both stable and highly variable environments.

Given the inherent difficulties in measuring root traits, the lack of cost-effective screening tools, and too little evidence of benefit if selections for specific root traits are made, roots have up to now garnered only little attention in breeding programmes. However, through high-throughput phenotyping tools like shovelomics (Trachsel *et al.*, 2011), X-ray computed tomography (Mooney *et al.*, 2012) and minirhizotrons, root trait screening has recently been advanced. Additionally, electromagnetic induction (EMI) surveying offers a non-invasive method to assess spatial soil variability and root–soil interactions in the field (Doolittle and Brevik, 2014; Whalley *et al.*, 2017). These technologies, combined with genomic selection approaches, are making the inclusion of below-ground traits in crop improvement more feasible and effective for future studies. Targeting root traits can lead to the development of more resilient wheat varieties capable of maintaining or improving yields under varying and increasingly harsh climatic conditions, such as drought and temperature fluctuations.

### Suitability of the study design

The field design of this study is notable for its scale and consistency: root samples were collected from the same set of ten wheat varieties on 11 European sites, covering a wide pedoclimatic gradient. This design enabled the collection of over 3500 root samples as well as diverse pedoclimatic and management data, providing a robust dataset for explorative correlation analyses. Compared with typical root phenotyping field studies, which often use only one or a few sites (Heinemann *et al.*, 2023), our dataset is unusually rich in environmental breadth. This facilitates the assessment of environmental influences using quantitative gradients of soil, climate and management variables, instead of treating site as nominal variable.

However, as data were collected in only one growing season, the geographical climate gradient needed to serve as a proxy for climate variation. Sampling was carried out using a standardized, machine-operated method by two trained teams, minimizing bias from personnel variability. Still, the timing of sampling – conducted after harvest – and the duration of sample transport and storage varied slightly across sites and may have influenced root integrity. Similarly, sample processing was consistent and machine-assisted, but the washing method and sieve size, while standardized, may not have been optimal for capturing full root system architecture (Livesley *et al.*, 1999; Pierret *et al.*, 2005). As a result, we focused on root length and surface area, which are more reliably preserved and measured than other root system architecture traits such as root growth angle or number of root tips under these sample processing conditions.

The use of two different upscaling approaches for topsoil and subsoil root traits is another limitation of our study. In the topsoil, upscaling to the area was not appropriate due to the small sample size per field plot (four crown root halves). In contrast, subsoil samples collected by soil coring could not be linked to individual wheat plants and therefore required upscaling to the soil surface area. This inconsistency prevented the calculation of total root length and surface area across the total soil depth of 1 m. As a result, our analysis focuses on the variability of root traits within individual soil depths.

Correlation analyses based on linear models are a valuable tool for exploring relationships between variables, but they have key limitations. Data transformations can distort results, especially when linearity is assumed without testing for non-linear patterns. These analyses often overlook interaction effects between predictors and risk overfitting when too many predictors are included. In agriculture, co-linearity among soil, climate and management variables (Supplementary Data Fig. S4) can complicate interpretation. To move towards causal understanding, future studies should use multi-year, site-replicated trials, alongside controlled experiments in which one factor is varied at a time.

## Supplementary Material

mcaf155_Supplementary_Data

## SUPPLEMENTARY DATA

Supplementary data are available at *Annals of Botany*  online and consist of the following. Detailed information on sampling, sample processing, and measurements is given in SIS1 and information on data analysis is given in SIS2. Supplementary tables are presented in SIS3 and supplementary figures in SIS4.
