# Supplementary material for: Linking root length and surface area to yield: variety-specific root plasticity in winter wheat across contrasting European environments
Source: Ann Bot. 2025 Jul 16;136(5-6):1219–37. doi: 10.1093/aob/mcaf155 (PMC12682866; doi:10.1093/aob/mcaf155)
Supplement: mcaf155_Supplementary_Data [file mcaf155_supplementary_data.pdf]

Supplementary information

SI1 Detailed information on sampling, sample processing and measurements

Root and soil sampling

The sampling was performed after wheat harvest in July and August 2022. At all sites except CH-Es, each of the three field replicates established for each variety was sampled once (total 30 plots per site). In CH-Es, the first replicate was sampled twice; those samples were considered as pseudo-replicates. For each sampling, two methods were used: (i) monolith excavation and (ii) soil coring. (i) One 0.25 x 0.25 x 0.15 m (L x W x D) soil monolith per plot comprising two wheat rows was excavated by driving a metal frame into the soil and retrieving the entire soil volume, from which all crown roots were collected. (ii) In addition, two soil cores per plot were taken with a soil auger (inner diameter 6 cm, outer diameter 8 cm), one directly on the crop row and one between the crop rows, up to a depth of 1.00 m. For the auger sampling, the sampling rod was loaded with a plastic liner and placed at the desired sampling location. The rod was driven into soil by means of an electronic impact hammer (Wacker EH50) and a mobile power generator, while the plastic liner on the inside unfolded gradually. After reaching a depth of 1 m, the rod was retrieved from the soil with a hydraulic pulling device (ZGM-9B Eco). The core was then retrieved from the rod and divided into five depth segments of 0.00–0.15 m, 0.15–0.30 m, 0.30–0.50 m, 0.50–0.75 m, and 0.75–1.00 m. Additionally, two extra soil cores were sampled from the centre of each field for the determination of soil characteristics. For the sampling period, samples were stored at ambient temperature for a maximum of ten days and then cooled at 4 °C for a maximum of 60 days or frozen at -18 °C for a maximum of one year.

Root sample processing and measurements

The crown roots were soaked in water to soften up the adhering soil and subsequently washed manually with a water jet (water pressure of approx. 35 N cm^{-2}) in a 500 µm sieve. After this step, the crown roots were separated from any remaining contaminants using a pair of tweezers. Subsoil samples were washed using a root washing machine (Hydropneumatic Elutriation System; Gillison's Variety Fabrication; Smucker et al. (1982)). The setup with eight independent cylinders allowed for efficient washing of the soil cores. The samples were fragmented into smaller pieces to ease the washing process and then washed for 15-50 min, depending on the soil type, with the air pressure set to 0.48 bar (7 psi on machine gauge) and water pressure set to 4 bar. The washed root samples were expelled into a 500 µm sieve by the machine; thereafter they had to be further processed by hand. For that, the content of the sieve was transferred into a smaller container filled with water and separated for wheat roots and remaining contaminants (mineral particles, husks, straw, roots of preceding crops) using a pair of tweezers (Hirte et al., 2017). Depending on the level of contamination, this step could take from 2 up to 30 minutes. The root samples of ES-Le had exceptionally high proportions of roots of the preceding crop alfalfa in all soil depths. Hence, those samples were not subjected to root measurements by scanning but were instead directly oven-dried and weighed.

For the root scanning, we used an Epson perfection v850 flat-bed scanner with a custom-made Plexiglas(R) tray (York, 2020), to allow for in-water scans of the roots. We scanned the samples in "photo mode", choosing "Transparency unit" as document source and "B&W Positive Film" as image type. We used a resolution of 600 dpi and set the scan quality to "high". To allow for easy 2D scanning of the crown roots, we cut the crown roots in two halves. From each sample, we took a subsample of two specimens, resulting in four crown root halves, placed them in the tray, and executed the scan on the dry samples. To scan the subsoil roots, we placed them into the tray, added water and carefully disentangled the roots inside the tray. Although we made sure that the roots were spread out over the entire scanning area, some overlap between

individual root parts was unavoidable. After scanning, the roots were placed into small aluminium containers, oven-dried at 40 °C for at least 24 h, and weighed on a micro balance (Mettler AT261 DeltaRange).

SI2 Detailed information on data analyses

Estimation of missing data for the row or inter-row position

A total of 69 values from all sampling depths of sites BE-Ge, CZ-Cr, DE-No and NO-As were missing for one sampling position only (36 on rows and 33 between rows). We calculated the proportion of row to inter-row value for each variety, site, and depth. This proportion was then used in a linear model to estimate the proportion per variety, site, and soil layer:

$$Y_i \sim \beta + \beta_1 S_{i1} + \beta_2 V_{i2} + \beta_3 D_{i3} + \varepsilon_i \quad (1)$$

where Y_i is the proportion, S_i is the site, V_i is the variety, D_i is the soil layer, and ε_i is the error term.

The value of the missing sampling position was then approximated by using the estimated marginal mean (emmean) of the proportion of row to inter-row value of this variety at the given site and depth.

$$root\ trait_{interrow\ upscaled} = \frac{root\ trait_{row\ upscaled}}{emmean\ prop\ root\ trait} - root\ trait_{row\ upscaled} \quad (2)$$

$$root\ trait_{row\ upscaled} = \frac{root\ trait_{interrow\ upscaled}}{emmean\ prop\ root\ trait} - root\ trait_{row\ upscaled} \quad (3)$$

where $root\ trait_{row\ upscaled}$ and $root\ trait_{interrow\ upscaled}$ are area-related root length [m root m^{-2} soil] or surface area [m^2 root m^{-2} soil] within and between rows, respectively, and

$emmean\, prop\, root\, trait$ is the estimated marginal mean of the proportion of row to interrow root length [m root m^{-2} soil] or root surface area [m^2 root m^{-2} soil] per variety.

Estimation of the relative importance of pedoclimatic and management variables for root length and surface area

The relative importance of all variables for root length and surface area was assessed using a random forest model:

$$\log\ (root\ trait) \sim pedoclimatic\ or\ management\ variable_1 + \cdots + pedoclimatic\ or\ management\ variable_{15} \tag{4}$$

The 15 variables were then ordered in decreasing order of importance based on the root mean square error loss after 50 permutations.

Model diagnostics

Table 5: After removing outliers of root length and surface area at the 95% CI (topsoil roots: 10 and 14 observations, 3 and 4.5% of total data; subsoil roots 0.15-0.50 m: 9 and 13 observations, 3 and 4.5% of total data; subsoil roots 0.50-1.00 m: 9 and 10 observations, 3.5 and 3.8% of total data), model diagnostics yielded no influential cases (Cook's distance > 1). The marginal R^2 ranged from 0.02-0.04 and the conditional R^2 ranged from 0.69-0.82.

Figure 2: After removing outliers at the 95% CI (6-8 observations, 3-4.5% of total data), model diagnostics yielded no influential cases (Cook's distance > 1) for any model. The marginal R^2 ranged from 0.02-0.10 and the conditional R^2 ranged from 0.79-0.88.

Figures 3-5: After removing outliers at the 95% CI (4-14 observations, 1.3-4.6% of total data), model diagnostics yielded no influential cases (Cook's distance > 1) for any model. The marginal R^2 ranged from 0.03-0.28 and the conditional R^2 ranged from 0.80-0.92.

Supplementary figures 6-8: After removing outliers at the 95% CI (topsoil roots: 7 and 18 observations, 2 and 5.8% of total data; subsoil roots 0.15-0.50 m: 9 and 13 observations, 3 and 4.5% of total data; subsoil roots 0.50-1.00 m: 7 and 11 observations, 2.7 and 4.2% of total data), model diagnostics yielded no influential cases (Cook's distance > 1). The marginal R^2 ranged from 0.02-0.68 and the conditional R^2 ranged from 0.31-0.86.

References

Hirte, J., Leifeld, J., Abiven, S., Oberholzer, H.-R., Hammelehle, A., & Mayer, J. (2017). Overestimation of Crop Root Biomass in Field Experiments Due to Extraneous Organic Matter. *Frontiers in Plant Science*, *8*. https://doi.org/10.3389/fpls.2017.00284

Smucker, A. J. M., McBurney, S. L., & Srivastava, A. K. (1982). Quantitative Separation of Roots from Compacted Soil Profiles by the Hydropneumatic Elutriation System1 [https://doi.org/10.2134/agronj1982.00021962007400030023x]. *Agronomy Journal*, *74*(3), 500-503. https://doi.org/https://doi.org/10.2134/agronj1982.00021962007400030023x

York, L. M. (2020). Plans for root scanning trays to use on flatbed scanners. *Zenodo*. https://doi.org/10.5281/zenodo.4122423

SI3 Supplementary tables

Supplementary table 1: Weather conditions for the 11 sites in the growing season 2021/22.

Site abbreviation	Temperature [°C]				Precipitation [mm]			
	Season	Emergence	Flowering	Harvest	Season	Emergence	Flowering	Harvest
AT-Gn	8.7	8.7	6.0	19.6	290	11	158	122
BE-Ge	9.6	8.2	6.8	16.9	459	46	287	126
CH-Ca	9.3	9.8	6.7	20.2	525	5	445	75
CH-Es	8.8	6.0	6.2	19.7	627	26	450	151
CZ-Cr	9.6	12.9	6.5	20.5	275	22	153	100
DE-Fr	8.1	6.4	5.2	18.9	511	20	339	152
DE-No	9.3	8.8	5.9	19.0	389	13	304	72
ES-Le	13.3	5.1	8.4	23.3	156	5	102	50
HU-Sz	8.6	3.1	6.8	20.4	331	58	89	185
LT-Do	7.2	9.7	3.5	18.7	676	24	388	265
NO-As	6.7	11.1	3.6	16.8	588	154	305	130

Supplementary table 2: Soil parameters for the 11 sites.

Site abbreviation	Clay content [%]			Bulk density [g cm^{-3}]			pH ($CaCl_2$) [-]			Total nitrogen [%]			Available phosphorus [mg kg^{-1}]			Total inorganic carbon [%]			Total organic carbon [%]		
	0.15 m	0.50 m	1.00 m	0.15 m	0.50 m	1.00 m	0.15 m	0.50 m	1.00 m	0.15 m	0.50 m	1.00 m	0.15 m	0.50 m	1.00 m	0.15 m	0.50 m	1.00 m	0.15 m	0.50 m	1.00 m
AT-Gn	28	28	22	1.0	1.1	1.2	7.5	7.6	7.7	0.2	0.1	0.1	25.1	9.5	1.6	0.6	1.6	3.5	2.2	1.8	0.6
BE-Ge	13	15	19	1.4	1.4	1.4	6.3	6.5	6.5	0.1	0.1	0.1	72.8	59.4	16.2	0.0	0.0	0.0	1.3	1.0	0.3
CH-Ca [1]	37	36	N/A	0.9	0.9	N/A	7.2	7.2	N/A	0.3	0.3	N/A	71.8	59.3	N/A	0.2	0.6	N/A	2.7	2.2	N/A
CH-Es	18	21	29	1.1	1.4	1.4	5.2	5.4	6.3	0.1	0.1	0.1	47.2	28.0	5.7	1.4	0.0	0.0	1.4	1.0	0.5
CZ-Cr	22	25	32	1.3	1.3	1.2	7.2	7.4	7.4	0.1	0.14	0.1	42.6	43.0	38.4	0.2	0.1	0.1	1.6	1.5	1.1
DE-Fr	20	29	27	1.3	1.5	1.7	6.6	6.2	5.5	0.1	0.04	0.0	40.5	18.6	8.8	0.0	0.0	0.0	1.3	0.5	0.2
DE-No	16	18	19	1.4	1.3	1.4	6.4	6.6	6.8	0.2	0.04	0.0	81.0	55.2	40.8	0.0	0.0	0.0	1.4	0.6	0.2
ES-Le [2]	24	23	16	1.0	1.2	1.3	7.5	7.55	7.6	0.2	0.1	0.1	38.0	17.0	8.3	5.1	5.6	N/A	2.3	1.3	N/A
HU-Sz	22	24	23	1.4	1.2	1.2	7.6	7.6	7.6	0.2	0.1	0.1	9.9	13.9	6.4	0.6	0.3	1.7	2.2	1.9	1.1

LT-Do	13	20	11	1.4	1.6	1.7	6.8	6.9	7.6	0.1	0.04	0.0	12.9	5.8	0.9	0.1	0.1	2.4	1.1	0.6	0.2
NO-As	21	25	15	1.2	1.3	1.6	5.2	5.0	5.1	0.2	0.2	0.1	30.4	18.8	7.9	0.0	0.0	0.0	2.3	1.6	0.7

[1] Sampling depth limited to 0.50 m

[2] Sampling depth limited to 0.75 m

Supplementary table 3: Analysis settings for RhizoVision Explorer.

Parameter	Subsoil roots	Topsoil roots	Topsoil roots separated
Analysis mode	Broken root	Whole root	Whole root
Convert pixels to physical unit	600 DPI	600 DPI	600 DPI
Image thresholding level	235	230	230
Keep largest component	-	false	true
Filter non root object	1 mm^2	8 mm^2	-
Fill holes in root objects	false	false	false
Enable edge smoothing	false	false	false
Root pruning threshold	5	10	10
Diameter ranges	0-0.25 / 0.25-0.5 / 0.5-1 / 1-2 / 2-4 / >4 mm	0-0.25 / 0.25-0.5 / 0.5-1 / 1-2 / 2-4 / >4 mm	0-0.25 / 0.25-0.5 / 0.5-1 / 1-2 / 2-4 / >4 mm

Supplementary table 4: Root parameters measured by RhizoVision Explorer in 'whole root' (topsoil roots) and 'broken root' (subsoil roots) modes (x measured; - not measured). Root parameters selected for this study are highlighted by a bold x.

Root parameter	Unit	Topsoil roots	Subsoil roots
Median and maximum number of roots	-	x	x
Number of root tips	-	x	x
Root length	mm	**x**	**x**
Perimeter	mm	x	x
Median diameter	mm	x	x
Average diameter, maximum diameter	mm	x	x
Volume	mm^2	x	x
Surface area	mm^3	**x**	**x**
Network area	mm^2	x	x
Branch points and branching frequency	-	-	x
Depth	mm	x	-
Maximum width	mm	x	-
Width-to-depth ratio	-	x	-
Convex area / solidity	mm^2 / -	x	-
Lower root area	mm^2	x	-
Number of holes / average hole size	- / mm^2	x	-
Average root orientation	°	x	-
Shallow angle frequency	-	x	-
Medium angle frequency	-	x	-
Steep angle frequency	-	x	-

SI4 Supplementary figures

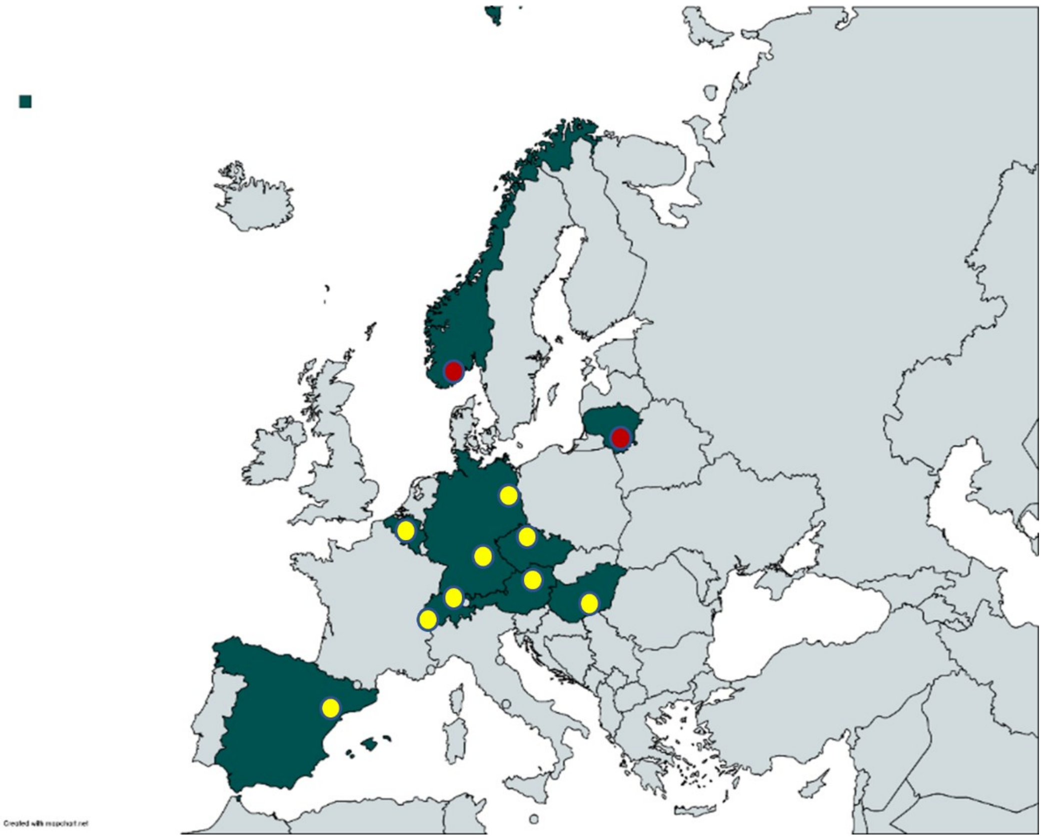

Supplementary figure 1: Map of Europe with winter wheat sites sampled in the project MaxRoot-C. Sites established as part of INVITE and MaxRoot-C are marked by yellow and red points, respectively.

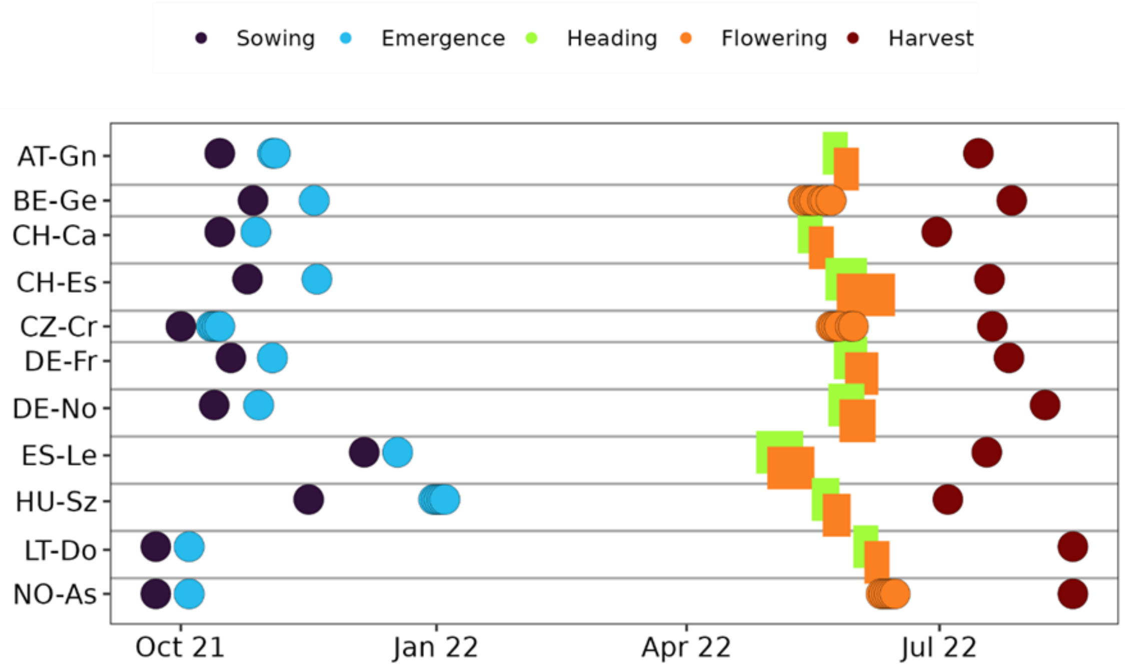

Supplementary figure 2: Approximate dates of crop management and phenology of ten winter wheat varieties at 11 sites in Europe in the wheat season 2021/22.

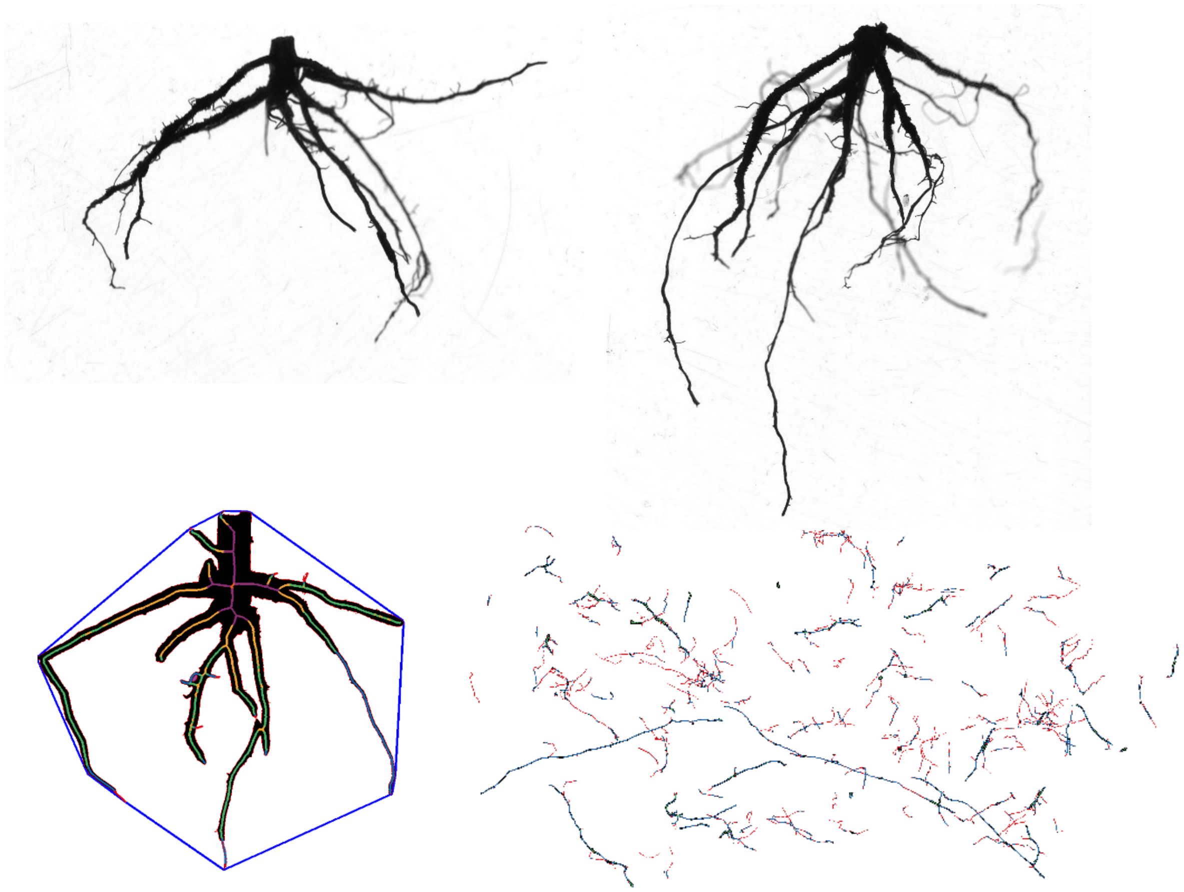

Supplementary figure 3: Root scans of two crown root halves (top left and top right), analysed crown root half (bottom left), and analysed subsoil roots (bottom right). Roots are coloured according to diameter ranges upon analysis with RhizoVision Explorer.

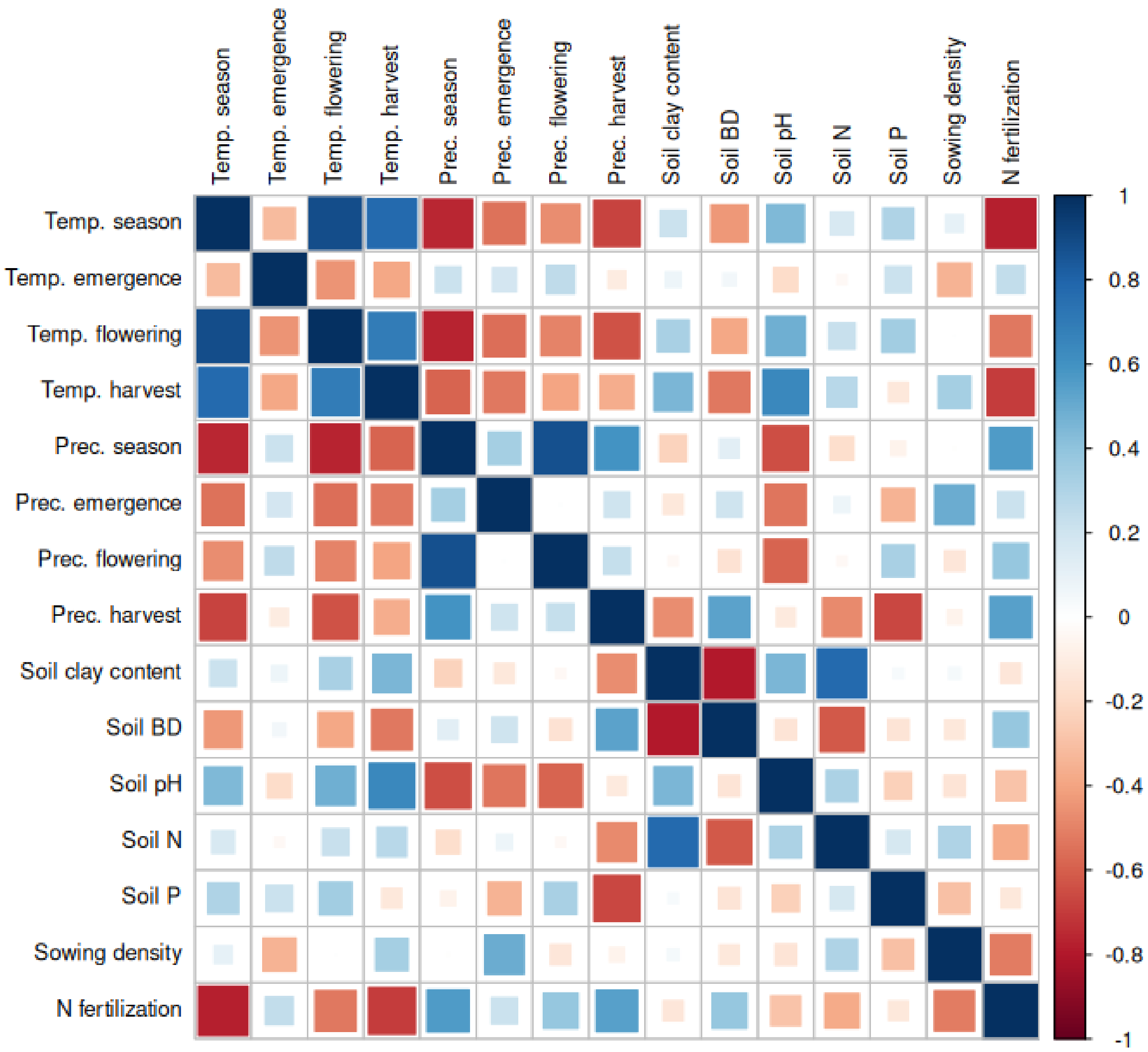

Supplementary figure 4: Multivariate Pearson correlation of the pedoclimatic and management variables of the 11 study sites. Soil variables refer to the topsoil (0.00-0.15 m).

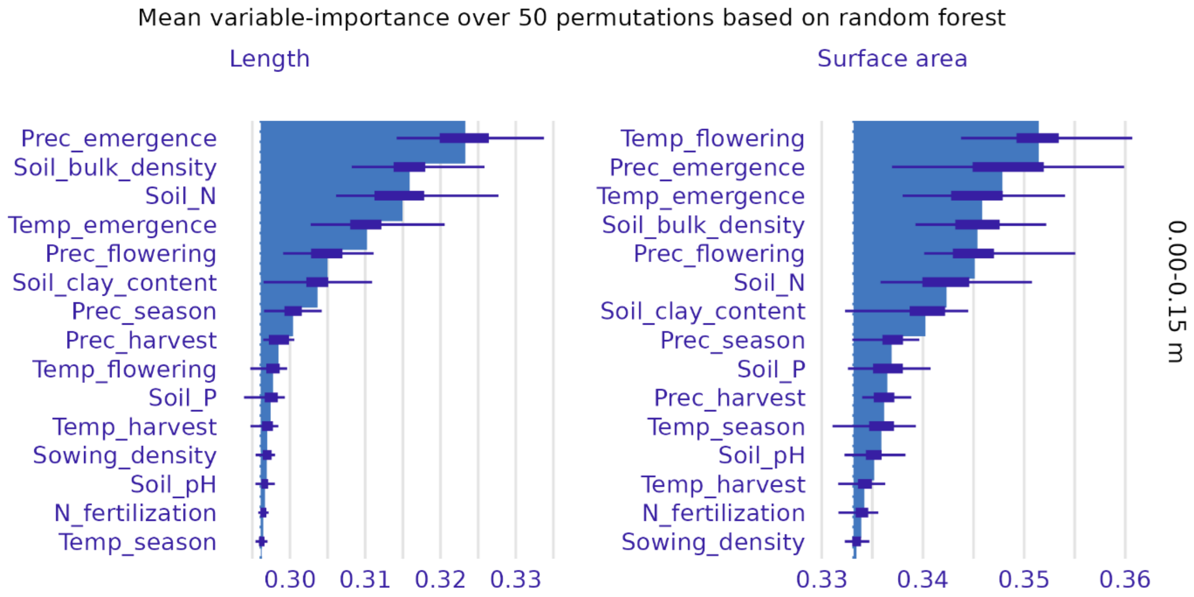

Supplementary figure 5: Variable importance of pedoclimatic and management variables for root traits of topsoil roots based on a random forest model analysis over 50 permutations.

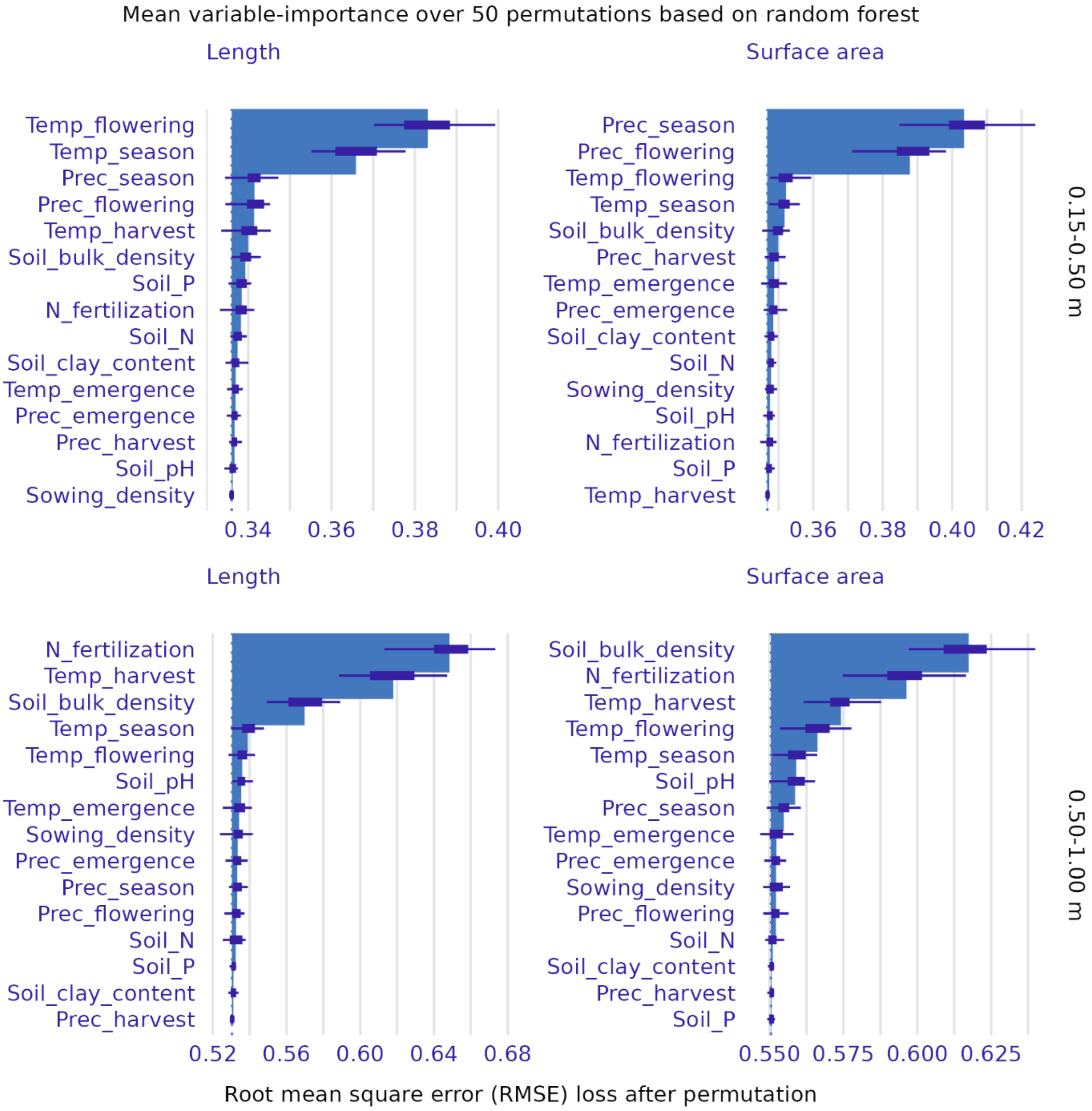

Supplementary figure 6: Variable importance of pedoclimatic and management variables for root traits of subsoil roots based on a random forest model analysis over 50 permutations.

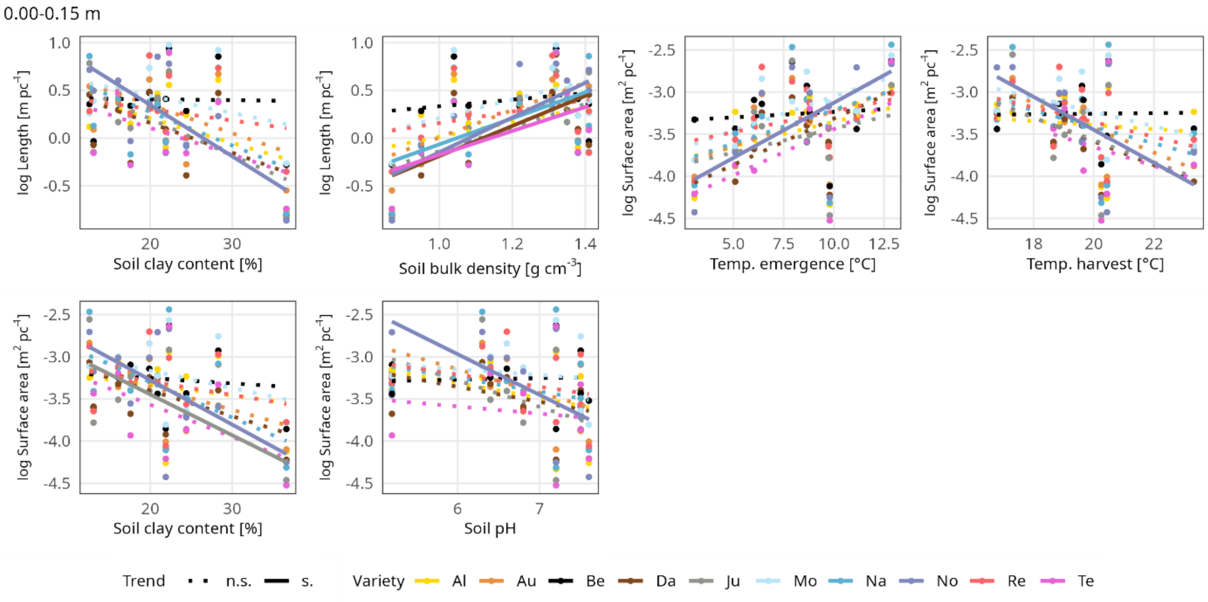

Supplementary figure 7: Relationship of pedoclimatic variables and root length and surface area in the topsoil. Points are average values across replicates per site. Trend lines are derived from mixed model output with site and replicate as nested random effects. n.s.: not significant, s.: significantly different from zero. Please refer to Table 3 for variety abbreviations

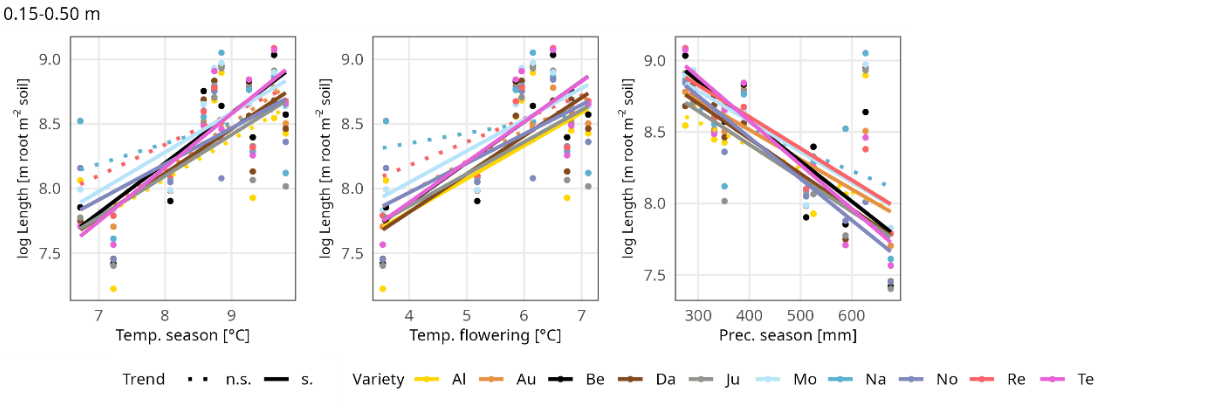

Supplementary figure 8: Relationship of pedoclimatic variables and root length in 0.15-0.50 m soil depth. Points are average values across replicates per site. Trend lines are derived from mixed model output with site and replicate as nested random effects. n.s.: not significant, s.: significantly different from zero. Please refer to Table 3 for variety abbreviations.

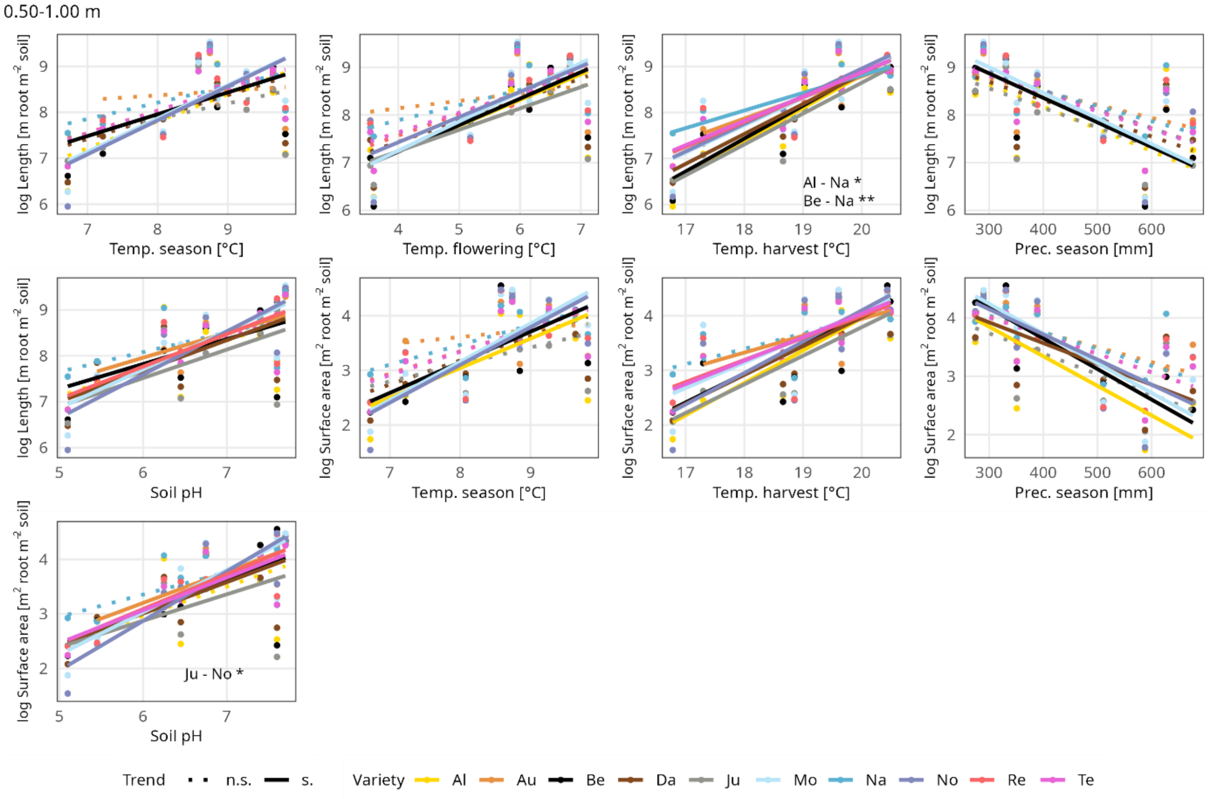

Supplementary figure 9: Relationship of pedoclimatic variables and root length and surface area in 0.50-1.00 m soil depth. Points are average values across replicates per site. Trend lines are derived from mixed model output with site and replicate as nested random effects. n.s.: not significant, s.: significantly different from zero. Slopes differing significantly from each other are indicated in the bottom right of the respective panel ($p < 0.05$, $p < 0.01$**, $p < 0.001$***). Please refer to Table 3 for variety abbreviations.*

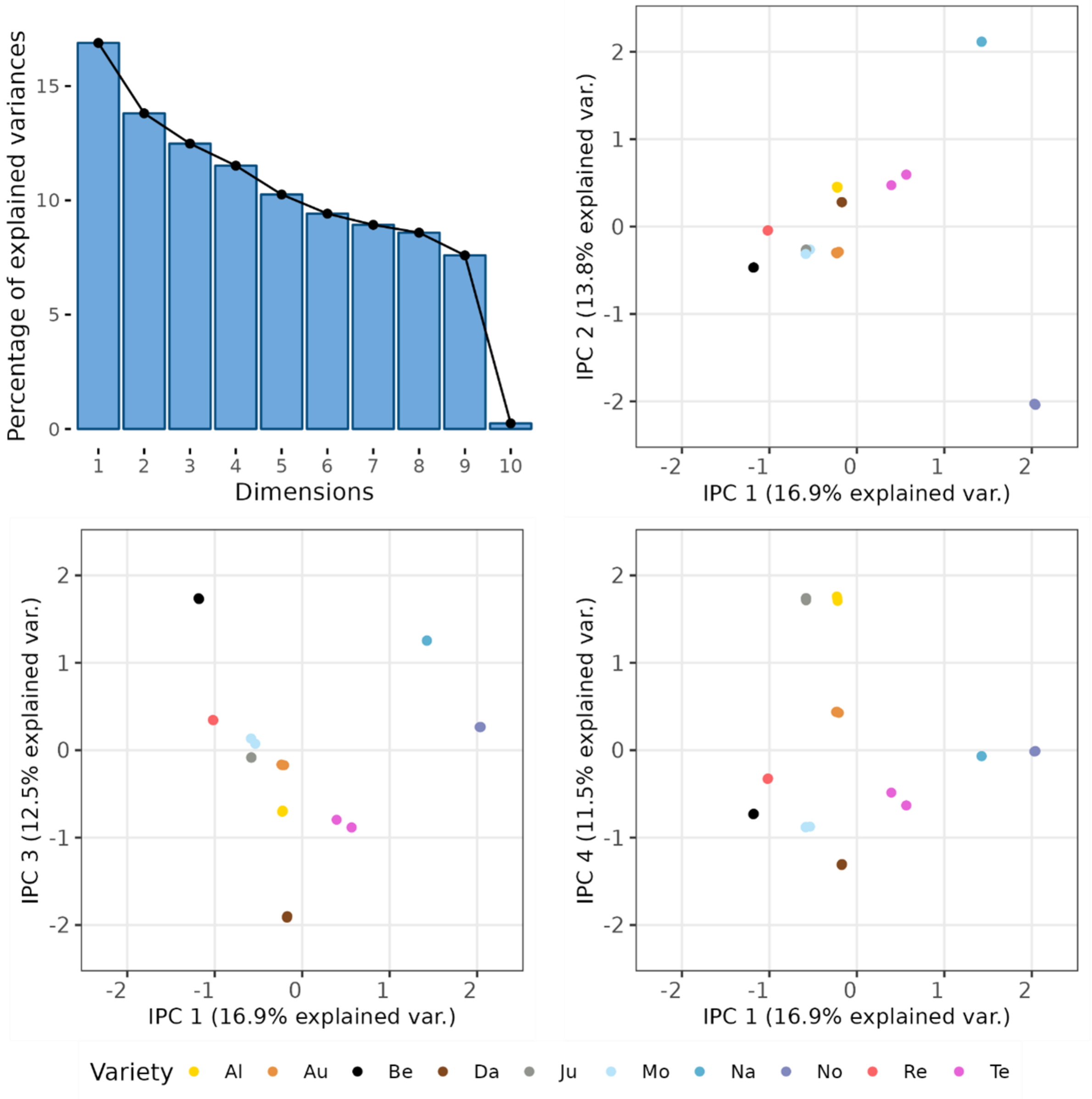

Supplementary figure 10: Double centered principal component analysis (DC-PCA) on the 25k SNP data grouped by variety. Scree plot indicating the percentage of explained variance by the first ten components (top left), IPC plot of the first and second interaction components (top right), the first and third components (bottom left) and the first and fourth components (bottom right). Please refer to Table 3 for variety abbreviations.

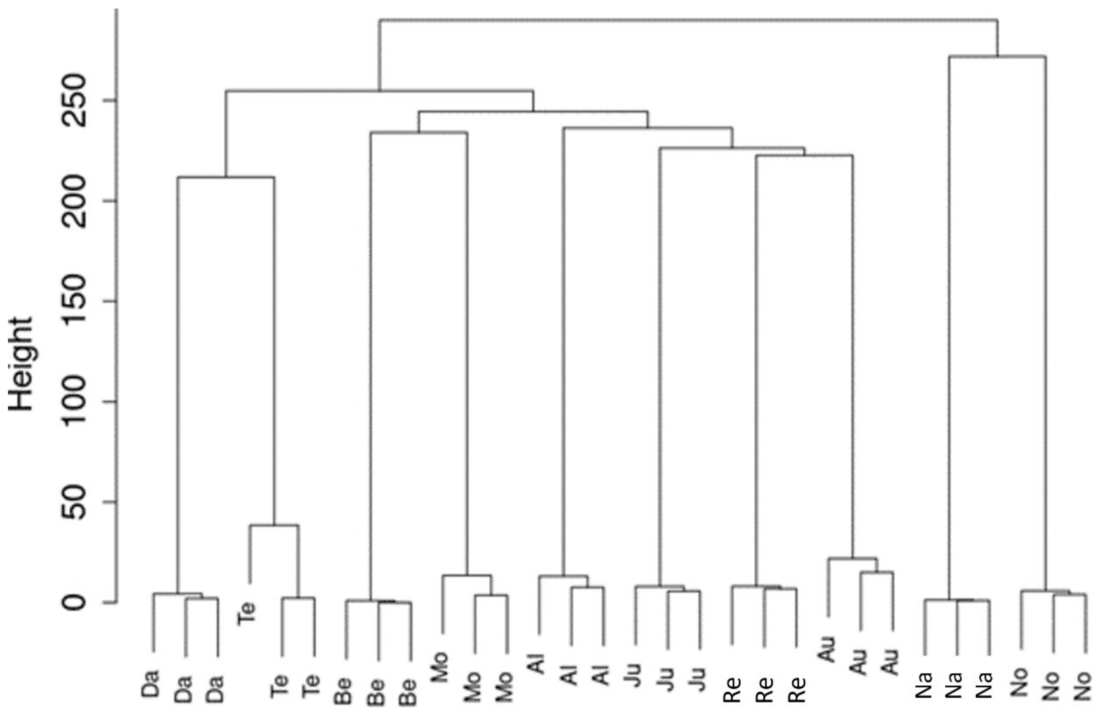

Supplementary figure 11: Cluster dendrogram of the ten wheat varieties based on 25k SNP data. Please refer to Table 2 for variety abbreviations.
